# The *E. coli* High-Pathogenicity Island Downregulates *PI3K*/*Akt*/*mTOR* Expression and Induces Autophagy in the Mouse Intestine

**DOI:** 10.3390/cells15151340

**Published:** 2026-07-26

**Authors:** Wen Li, Bo Zhang, Weiwei Zhao, Hao Wang, Meng Zhou, Yue Li, Jinzhi Ma, Leyi Chu, Xiaofeng Ruan, Peng Xiao, Hong Gao

**Affiliations:** 1 College of Animal Science and Technology, Yunnan Agricultural University, Kunming 650201, China; liwen5212022@163.com (W.L.); zhangbosicau@126.com (B.Z.); 2 College of Food Science and Technology, Yunnan Agricultural University, Kunming 650201, China; zww@dali.edu.cn (W.Z.); wanghaoxu0117@163.com (H.W.); zhoumeng@mail.kiz.ac.cn (M.Z.); 3 College of Veterinary Medicine, Yunnan Agricultural University, Kunming 650201, China; 18184868232@163.com (Y.L.); 15108713682@163.com (J.M.); 16691511254@163.com (L.C.); 18214107133@163.com (X.R.)

**Keywords:** *Escherichia coli*, high-pathogenicity island (HPI), autophagy, PI3K/Akt/mTOR pathway, macrophages

## Abstract

The high-pathogenicity island (HPI) is a major virulence determinant in pathogenic *Escherichia coli* (*E. coli*), contributing to severe inflammation and tissue damage. Autophagy plays a critical role in clearing intracellular pathogens and modulating inflammation, but whether HPI manipulates this process remains unknown. Here, using a swine-pathogenic *E. coli* strain and its HPI-deficient mutant (Δirp2) generated by CRISPR/Cas9, we investigated the interplay between HPI and autophagy in RAW264.7 macrophages and a mouse intestinal infection model. We found that HPI^+^ infection induced autophagic activation, as evidenced by increased LC3 puncta (immunofluorescence), upregulated *Beclin-1* and autophagy-related gene mRNA levels (qPCR), and downregulated *phosphatidylinositol 3-kinase (PI3K)*/*protein kinase B (Akt)*/*mammalian target of rapamycin (mTOR)* expression at both mRNA (qPCR) and protein (immunohistochemistry) levels. In a mouse model, HPI^+^ infection upregulated intestinal Microfold (M) cell markers and secretory Immunoglobulin A (IgA), triggered robust production of pro-inflammatory cytokines, and induced more severe tissue pathology than the HPI-deficient mutant. Pharmacological activation of autophagy with rapamycin alleviated HPI-induced inflammation and injury, whereas inhibition of autophagy by 3-methyladenine (3-MA) or *Beclin-1* silencing exacerbated damage. These findings suggest that HPI induces autophagy, but the endogenous autophagic response is insufficient to counteract HPI-induced pathology; pharmacological enhancement of autophagy partially alleviated this insufficiency and reduced tissue damage. Notably, *Beclin-1* knockdown blunted HPI-induced upregulation of *PI3K* and autophagy-related genes, suggesting a role for *Beclin-1* in the transcriptional regulation of these responses. In conclusion, HPI simultaneously exerts direct pro-inflammatory effects and induces *Beclin-1*-dependent autophagy. Enhancing this autophagic response pharmacologically, rather than relying on the endogenous level triggered by HPI alone, limits excessive tissue damage. Thus, boosting autophagy may represent a promising therapeutic strategy against HPI-bearing pathogenic *E. coli* infections.

## 1. Introduction

*Escherichia coli* (*E. coli*), a member of the Enterobacteriaceae family (phylum Proteobacteria), typically represents only a small proportion of the commensal gut microbiota [1]. However, *E. coli* is among the most common pathogens causing a broad range of intestinal and extraintestinal infections [2]. Host factors such as inflammation can disrupt the native colonic microbial community, fostering a niche that favors the expansion of resident or introduced Enterobacteriaceae, including *E. coli* [3]. The high-pathogenicity island (HPI), which encodes the *yersiniabactin* siderophore system for iron acquisition, is a key virulence determinant initially identified in *Yersinia* spp. and now widely disseminated among pathogenic Enterobacteriaceae, including *E. coli*. In *E. coli*, HPI enhances bacterial fitness, motility, and virulence, in part by regulating siderophore metabolism [4]. The HPI confers a significant survival advantage by enabling the production of *yersiniabactin*, a metallophore that chelates iron, copper, and zinc to support infection under varying host conditions. This mobile virulence island can spread to *E. coli* via horizontal gene transfer. Its successful dissemination and function are shaped by a composite genetic architecture, featuring both highly conserved core sequences and adaptive, variable components [5]. Collectively, HPI has pleiotropic effects that contribute to its multifaceted role in *E. coli* virulence. However, whether HPI interacts with host cell autophagy to influence infection outcomes remains unknown.

Microfold (M) cells serve as a primary route by which many pathogens breach the mucosal barrier. A variety of pathogenic microorganisms can adhere to M cells and, following transcytosis, induce local mucosal inflammation and systemic infection [6]. Upon infection, M cells transport luminal antigens to the underlying Peyer’s patches, where antigen-presenting cells process the antigens and activate B cells to initiate humoral immune responses [7]. The activated B cells differentiate into plasma cells, which produce and secrete Immunoglobulin A (IgA). IgA then associates with the secretory component expressed on intestinal epithelial cells to form secretory IgA (sIgA), which is subsequently released into the intestinal lumen. sIgA can bind to the apical surface of M cells, further facilitating antigen uptake and enhancing the induction of immune responses. In addition to their role in antigen sampling, M cells have also been shown to transport luminal sIgA to subepithelial lymphoid follicles and deliver it to dendritic cells, thereby contributing to the modulation of mucosal immune responses [8]. M cells thus play a pivotal role in regulating the intestinal mucosal immune system and are closely linked to the maintenance of lymphocyte homeostasis, the structural integrity of Peyer’s patches, and the regulation of lymphocyte activation. Beyond the mucosal immune regulation mediated by M cells, autophagy represents another key host defense mechanism that restricts bacterial survival and modulates inflammatory responses.

Autophagy is a conserved lysosomal degradation pathway essential for cellular homeostasis, responsible for clearing damaged organelles and misfolded proteins [9]. As a cell-autonomous cytoplasmic process, autophagy is regulated by nutrient and growth sensing pathways, including mammalian target of rapamycin (mTOR) [10], adenosine 5′-monophosphate-activated protein kinase (AMPK) [11], and insulin signaling. Autophagy is commonly classified into macroautophagy, microautophagy, and chaperone-mediated autophagy based on how cargo is delivered to lysosomes [12,13]. Macroautophagy, the most extensively studied type, entails the de novo formation of double-membraned autophagosomes to sequester cytoplasmic material, a process driven by a conserved set of Autophagy-related (ATG) proteins [14]. Among more than 35 ATG proteins, microtubule-associated protein light chain 3 (LC3) is integral to autophagosome biogenesis and serves as a gold standard marker for monitoring autophagic flux [15]. Beyond cellular housekeeping, autophagy also functions as an important effector of innate immunity. Macrophages, as professional phagocytes, leverage autophagy to eliminate intracellular pathogens and restrain bacterial replication, thereby preventing the establishment of a protected niche for microbial survival. Autophagic activity in macrophages can be strongly induced by bacterial infection and inflammatory cues. Notably, inflammatory host responses in the gut can reshape the microbial landscape, favoring the expansion of facultative anaerobes such as Enterobacteriaceae [16]. Given that macrophages are key effectors against bacterial infection, whether macrophage autophagy promotes clearance of phagocytosed *E. coli* HPI-positive (*E. coli* HPI^+^) remains unclear.

The phosphatidylinositol 3-kinase (PI3K)/protein kinase B (Akt)/mTOR pathway is a major regulator of autophagy and contributes to the pathogenesis of many diseases [17]. Autophagy is differentially regulated by class I and III PI3K enzymes within the broader PI3K/Akt/mTOR pathway. Class I PI3K signaling generally suppresses autophagy. Conversely, the class III enzyme VPS34 complexes with Beclin-1 and phosphoinositide-3-kinase regulatory subunit 4 (*PIK3R4*) to produce phosphatidylinositol 3-phosphate (PI3P), a key lipid required for autophagosome formation and the progression of autophagy. Activation of growth-factor receptors promotes PI3K recruitment and activation. These enzymes subsequently generate 3′-phosphoinositide lipids, which function as critical second messengers by recruiting and activating a diverse array of downstream cellular target proteins [18]. Akt, a serine/threonine kinase with three major isoforms, is a key downstream effector. PI3Ks themselves act as principal signaling effectors downstream of receptors such as G protein-coupled receptors (GPCRs) and receptor tyrosine kinases (RTKs), transducing extracellular signals from cytokines and growth factors into intracellular instructions via phospholipid production; these lipid products then stimulate Akt along with other effector pathways [19]. A principal function of Akt is to activate the rapamycin-sensitive mTOR complex 1 (mTORC1). Furthermore, the rapamycin-insensitive mTOR complex 2 (mTORC2) contributes to pathway regulation by phosphorylating Akt on a critical residue, thereby illustrating a tightly interconnected relationship between PI3K and mTOR signaling [20]. In this pathway, mTOR serves as a central inhibitory checkpoint for autophagy. Accordingly, pharmacological or genetic inhibition of the PI3K/Akt/mTOR axis can induce autophagy. However, the involvement of the PI3K/Akt/mTOR pathway in *E. coli* HPI infection remains to be fully characterized.

Here, we investigated autophagy responses during *E. coli* HPI^+^ infection in mice and macrophages, with a focus on intestinal M cell and tissue responses, to clarify how these responses contribute to bacterial pathogenicity.

## 2. Materials and Methods

### 2.1. Bacterial Culture and Recovery Experiment

The enteropathogenic *E. coli* HPI^+^ used in this study was originally isolated from a Yunnan Saba pig farm in Chuxiong Prefecture where piglets had been diagnosed with yellow dysentery. This strain has been assigned the GenBank accession number JAEANZ000000000. The corresponding HPI-negative mutant (*E. coli* ΔHPI) was generated in a previous study via CRISPR/Cas9-mediated gene editing [21]. The cryopreserved wild-type *E. coli* HPI^+^ strain and the CRISPR/Cas9-generated *E. coli* ΔHPI strain were recovered on MacConkey agar (Huankai Microbial Sci. & Tech. Co., Ltd., Guangzhou, China, 022143) and incubated at 37 °C for 18 h. Single colonies were then picked using an inoculating loop and transferred into liquid LB medium, followed by incubation with shaking at 37 °C until the OD600 reached 0.6–0.8. The bacterial cultures were subsequently stored at 4 °C for further use.

### 2.2. Mice

Kunming mice (5 weeks old, weighing 24 ± 2 g) were purchased from Kunming Medical University (License No.: SCXK (Dian) K2015-0002). The mice were housed in the Animal Pathology Laboratory of Yunnan Agricultural University under controlled conditions: temperature 25 ± 2 °C, humidity 40 ± 2%, ad libitum access to food and water, and a 12 h light/dark cycle.

Mice were challenged with either the *E. coli* HPI^+^ or *E. coli* ΔHPI strain via intraperitoneal injection at a dose of 100 μL per mouse (*n* = 5 per group). The experimental groups included the *E. coli* HPI^+^-infected group, the *E. coli* ΔHPI-infected group, and a control group (injected with an equal volume of sterile PBS). Mice from both bacterial challenge groups were euthanized at 6 h, 12 h, 18 h, 24 h, and 48 h post-infection for sample collection. Clinical manifestations were monitored continuously throughout the experiment, and pathological changes were recorded during necropsy.

Rapamycin and 3-methyladenine (3-MA) are well-established and widely recognized pharmacological tools in autophagy research. They modulate autophagy through opposing mechanisms: rapamycin acts as a specific inducer of autophagy, whereas 3-MA serves as a classical inhibitor. To investigate the functional role of autophagy in *E. coli* HPI infection, rapamycin and 3-MA were selected as pharmacological tools. Mice were administered rapamycin (Yuanye Bio-Technology Co., Ltd., Shanghai, China, B20714) (3 mg/kg) via intraperitoneal injection in the morning. The rapamycin powder was dissolved in dimethyl sulfoxide (DMSO) (Amresco, LLC, Solon, OH, USA, 0231) according to the manufacturer’s instructions. Twenty-four hours after the first rapamycin injection, a second dose was administered, followed 2 h later by an injection of 100 μL bacterial suspension (OD600 = 0.6–0.8). At 18 h post-bacterial challenge, a third dose of rapamycin was given, and mice were euthanized 2 h later. Intestinal tissues were collected and either frozen or fixed for subsequent analysis.

In parallel, 3-MA (Yuanye Bio-Technology Co., Ltd., Shanghai, China, S24823) was dissolved in sterile phosphate-buffered saline (PBS) (Yuanye Bio-Technology Co., Ltd., Shanghai, China, R20857) according to the manufacturer’s instructions and administered using the same injection schedule at a dose of 15 mg/kg. The experimental groups were designed as follows: Control group (PBS), *E. coli* HPI^+^ infection group, *E. coli* ΔHPI infection group, *E. coli* HPI^+^ + 3-MA group, *E. coli* ΔHPI + 3-MA group, *E. coli* HPI^+^ + rapamycin group, and *E. coli* ΔHPI + rapamycin group.

### 2.3. Cell Culture

RAW264.7 cells were obtained from the Kunming Institute of Zoology, Chinese Academy of Sciences (Kunming, China). The cells were rapidly thawed in a 37 °C water bath and transferred into 5 mL complete DMEM (Hyclone Laboratories, Inc., Logan, UT, USA, SH30022.01) supplemented with 10% fetal bovine serum (FBS) (Gibco, Inc., Waltham, MA, USA, A5670501), 100 U/mL penicillin, and 100 μg/mL streptomycin (Hyclone Laboratories, Inc., Logan, UT, SV30010). After centrifugation at 1000 rpm for 5 min, the cell pellet was resuspended in fresh complete medium and cultured in T25 flasks at 37 °C under 5% CO_2_. For subculture, cells at 80–90% confluence were washed with PBS, detached using 0.25% trypsin-0.53 mM EDTA (Gibco, Inc., Waltham, MA, USA, 25200056), and neutralized with complete medium. The cell suspension was split at a ratio of 1:2 or 1:3, and the culture was continued. For cryopreservation, harvested cells were resuspended in freezing medium (90% complete medium containing 10% DMSO) at a density of 1–3 × 10^6^ cells/mL, aliquoted into cryovials, and stored at −80 °C for 48 h before transfer to liquid nitrogen.

### 2.4. siRNA Synthesis and Cell Transfection

An siRNA targeting mouse macrophage *Beclin-1* was designed based on its mRNA sequence from NCBI using Oligo software (version 7.60, Molecular Biology Insights, Inc., Colorado Springs, CO, USA). All primers were synthesized by Sangon Biotech (Shanghai, China), with the following sequences:

Forward: 5′-CCUGGAUCGUGUUUACCAUUTT-3′

Reverse: 5′-AAUGGUAACACGAUCCAGGTT-3′

RAW264.7 cells were transfected as follows. Cells were seeded in 24-well plates at approximately 20% confluence one day before transfection. For each well, 20 pmol of siRNA was diluted in 12.5 μL of RNase-free water and gently mixed. Separately, 1 μL of Lipofectamine RNAiMAX transfection reagent (Thermo Fisher Scientific, Waltham, MA, USA, CMAX00008) was combined with 11.5 μL RNase-free water. The two solutions were then mixed, incubated at room temperature for 5 min, and added to the cells. The experimental groups included: Control group (cells treated with transfection reagent only), *E. coli* HPI^+^ infection, *E. coli* ΔHPI infection, *E. coli* HPI^+^ + si*Beclin-1*, *E. coli* ΔHPI + si*Beclin-1*. Samples were analyzed at 2 h, 4 h, 8 h, 12 h, and 16 h post-infection.

### 2.5. Cell Infection

Prior to bacterial infection, RAW264.7 cells were detached with trypsin, resuspended, and counted. An appropriate number of cells were seeded into culture plates and incubated for 16–24 h until approximately 80% confluence was reached. Before infection, the medium was aspirated, and cells were washed twice with PBS, followed by the addition of DMEM without serum or antibiotics. Bacterial suspensions were added at the designated multiplicity of infection (MOI), and infected cells were incubated at 37 °C under 5% CO_2_ until the specified time points. Unless otherwise specified, bacterial infections were performed at an MOI of 10, corresponding to approximately 1 × 10^6^ CFU of *E. coli* per well for RAW264.7 cells seeded at 1 × 10^5^ cells per well in 24-well plates; the bacterial inoculum was verified by serial dilution and plating on LB agar prior to each experiment. The following experimental groups were established, and inhibitor pretreatments were carried out as described below.

Experimental group one: Control (no infection), *E. coli* HPI^+^ and ΔHPI (at indicated MOI).

Experimental group two: Control (no infection), Rapamycin (Rapa; 3 μM, 12 h), *E. coli* HPI^+^ and ΔHPI (at indicated MOI).

Experimental group three: Control (no infection), *E. coli* HPI^+^ and ΔHPI (at specified MOI), Chloroquine (CQ; 50 μM, 6 h) (Yuanye Bio-Technology Co., Ltd., Shanghai, China, T94102 ) + HPI^+^, CQ + ΔHPI.

Experimental group four: Control (no infection), *E. coli* HPI^+^ and ΔHPI (at indicated MOI), LY294002 (LY: 10 μM, 1 h) (Yuanye Bio-Technology Co., Ltd., Shanghai, China, S43088) + HPI^+^, LY + ΔHPI, Triciribine (TCN, 10 μM, 1 h) (Yuanye Bio-Technology Co., Ltd., Shanghai, China, S80679) + HPI^+^, TCN + ΔHPI.

Experimental group five: Control (no infection), *E. coli* HPI^+^ and ΔHPI (at indicated MOI), Rapa (3 μM, 12 h) + HPI^+^, Rapa + ΔHPI, 3-MA (3 mM, 3 h) + HPI^+^, 3-MA + ΔHPI.

### 2.6. Phagocytosis Assay

RAW264.7 cells were detached from culture flasks using trypsin, resuspended by pipetting, counted with a cell counter, seeded in 24-well plates at a density of 3–4 × 10^5^ cells per well, and cultured in complete medium for 16–24 h until approximately 80% confluence was reached. Prior to bacterial infection, the medium was aspirated, and cells were washed twice with PBS, followed by the addition of DMEM without serum or antibiotics. Bacterial suspensions were then added at the indicated MOI, and the cells were incubated at 37 °C under 5% CO_2_ for the specified time to allow phagocytosis. After the co-incubation period, the cells were washed twice with PBS to remove uninternalized bacteria, followed by incubation with DMEM containing 100 μg/mL gentamicin (Solarbio Science & Technology Co., Ltd., Beijing, China, SG8690) for 1 h at 37 °C to eliminate extracellular bacteria. The cells were then lysed with 0.25% Triton X-100 (Servicebio Technology Co., Ltd., Wuhan, China, G3068) for 10 min at 37 °C. The lysates were serially diluted, plated onto LB agar plates, and incubated overnight at 37 °C. Bacterial colonies were enumerated, and the number of intracellular bacteria per well was calculated based on the dilution factor.

### 2.7. Cell Counting Kit 8 Assay

For the Cell Counting Kit 8 (CCK-8) assay (LABLEAD Biotechnology Co., Ltd., Beijing, China, CK001), RAW264.7 cells were seeded in 96-well plates at 8.0 × 10^4^ cells per well and cultured for 16–24 h. The cells were washed with PBS and incubated with 200 μL DMEM containing 20 μL CCK-8 reagent per well. Blank controls contained medium and CCK-8 only. Following a 1 h incubation at 37 °C, absorbance was measured at 450 nm and the cell viability was calculated.

### 2.8. Cytotoxicity Assay

Lactate dehydrogenase (LDH) cytotoxicity was determined using a Beyotime assay kit (Shanghai, China, C0016) according to the manufacturer’s instructions. RAW264.7 cells were seeded at 8.0 × 10^4^ cells per well in 96-well plates and cultured to 80–90% confluence. Before bacterial treatment, cells were switched to phenol red-free, serum-free DMEM. Experimental groups consisted of control (medium only), negative control (untreated cells), lysed cells (maximum LDH release), and bacteria-infected cells. The maximum release control wells received LDH release reagent 1 h before harvesting. Supernatants were centrifuged and analyzed by measuring absorbance at 490 nm with 600 nm as reference. Cytotoxicity was calculated as a percentage of maximum LDH release.

### 2.9. Hematoxylin & Eosin Staining

Mouse intestinal tissues were fixed in 4% paraformaldehyde (Baishi Chemical Co., Ltd., Tianjin, China, 30525-89-4), embedded in paraffin (Leica Biosystems, Deer Park, IL, USA, 39601006), and sectioned. Sections were deparaffinized in xylene (Youning Biotechnology Co., Ltd., Shanghai, China, abs42002437), rehydrated, and stained with hematoxylin (Solarbio Science & Technology Co., Ltd., Beijing, China, G1120). After differentiation and bluing in tap water, sections were dehydrated, counterstained with eosin (Solarbio Science & Technology Co., Ltd., Beijing, China, G1120), dehydrated again, cleared in xylene, and mounted with neutral balsam (Shanghai Specimen Model Factory, Shanghai, China, S3006).

RAW264.7 cells (with or without *Beclin-1* silencing) were cultured on coverslips, infected with the respective *E. coli* strains, and processed for hematoxylin & eosin staining. After PBS washing, cells were fixed with 4% paraformaldehyde, stained with hematoxylin for 10 min, differentiated in acid-alcohol, and counterstained with eosin for 2 min. Coverslips were then dehydrated through an ethanol series, cleared in xylene, and mounted with neutral balsam. All samples were examined under a microscope for pathological assessment.

### 2.10. RT-qPCR

Total RNA (Vazyme Biotech Co., Ltd., Nanjing, China, R711-01) was extracted from mouse intestinal tissues or RAW264.7 cells using a commercial RNA isolation kit, and genomic DNA was removed with a gDNA Eraser kit. cDNA was synthesized by reverse transcription using a PrimeScript RT reagent kit (TaKaRa Bio Co., Ltd., Dalian, China, RR047A). Quantitative PCR was performed in triplicate using SYBR Premix Ex Taq II (TaKaRa Bio Co., Ltd., Dalian, China, RR820A) on a real-time PCR system under the following conditions: 95 °C for 30 s, followed by 35 cycles of 95 °C for 5 s and 60 °C for 30 s, with a final melting curve analysis. The relative mRNA expression levels of target genes (including receptor activator of nuclear factor-κB ligand (RANKL), autophagy-related genes, and inflammatory cytokines) were normalized to β-actin and calculated using the 2^−ΔΔCt^ method. Primer sequences are listed in Table 1.

### 2.11. Immunohistochemical Analysis

For immunohistochemical analysis of mouse intestinal tissues, paraffin-embedded duodenal sections were deparaffinized, rehydrated, and subjected to antigen retrieval in citrate buffer (Yuanye Bio-Technology Co., Ltd., Shanghai, China, R26389). For immunocytochemical staining of cultured RAW264.7 cells, cells grown on coverslips were fixed, permeabilized, and blocked. Samples (both tissue sections and cell coverslips) were then incubated with specific primary antibodies: tissue sections with anti-M cell (1:100, Novus Biologicals, NBP1-86061), anti-IgA (1:200, Novus Biologicals, NB7506), anti-PI3K (1:600, Novus Biologicals, NBP1-89731), anti-Akt (1:500, Novus Biologicals, NBP3-48751), and anti-mTOR (1:800, Novus Biologicals, NB100-240); cell coverslips with anti-LC3 (1:1000, Novus Biologicals, NBP3-03280) and anti-Beclin-1 (1:1000, Novus Biologicals, NB500-249). All samples were subsequently incubated with an HRP-conjugated secondary antibody (Novus Biologicals, NBP1-72732H). Signal was developed using DAB (Maixin Biotech, Ltd., Fuzhou, China, KIT-0014). Tissue sections were then counterstained with hematoxylin, dehydrated, cleared, and mounted. Images were acquired microscopically and analyzed quantitatively with ImageJ software (version 1.53k, National Institutes of Health, Bethesda, MD, USA).

### 2.12. Immunofluorescence Analysis

Immunofluorescence was performed to assess the expression and subcellular localization of LC3 in both mouse duodenal tissues and RAW264.7 cells. Paraffin-embedded intestinal sections were deparaffinized, rehydrated, and subjected to antigen retrieval in citrate buffer, while cultured cells on coverslips were fixed with 4% paraformaldehyde and permeabilized with Triton X-100. After blocking with bovine serum albumin (BSA) (Labgic Technology Co., Ltd., Beijing, China, BS114), samples were incubated with a primary antibody against LC3 (1:200) (Proteintech Group, Inc., Rosemont, IL, USA, 14600-1-AP) followed by a fluorophore-conjugated secondary antibody (Proteintech Group, Inc., Rosemont, IL, USA, RGAR005). Nuclei were counterstained with DAPI (Servicebio Technology Co., Ltd., Wuhan, China, G1012). Images were acquired using an Olympus FV1000 confocal microscope with FV10-ASW software (version 3.1; Olympus, Center Valley, PA, USA).

### 2.13. ELISA

To assess intestinal immune responses, mice were infected with *E. coli* strains (with or without HPI gene deletion), and duodenal tissues were collected at 6, 12, 24, and 48 h post-infection. Tissue levels of interleukin-1β (IL-1β) (PI301), tumor necrosis factor-α (TNF-α) (PT513), and interferon-γ (IFN-γ) (PI507) were assayed using commercial ELISA kits (Beyotime Biotechnology, Shanghai, China) according to the manufacturer’s protocols. Similarly, RAW264.7 cells (including *Beclin-1* silenced groups) were infected, and cytokine levels (TNF-α, IL-1β, IFN-γ) in supernatants were determined by ELISA at 2, 4, 8, 12 and 16 h post-infection.

### 2.14. Transmission Electron Microscopy

The ultrastructural features of mouse duodenal tissues and infected RAW264.7 cells were examined by transmission electron microscopy (TEM). Briefly, samples were fixed with 2.5% glutaraldehyde (Servicebio Technology Co., Ltd., Wuhan, China, G1102), post-fixed in 1% osmium tetroxide (Sinopharm Chemical Reagent Co., Ltd., Shanghai, China, S4194941G), and dehydrated through a graded ethanol series. After embedding and polymerization, ultrathin sections were prepared and stained with 3% uranyl acetate (Electron Microscopy Sciences, Hatfield, PA, USA, 22400) and lead citrate (Electron Microscopy Sciences, Hatfield, PA, USA, 22410). Images were acquired using a JEM 100CXII (JEOL Ltd., Tokyo, Japan) transmission electron microscope.

### 2.15. Statistical Analysis

All data are presented as mean ± standard deviation (SD). Animal experiments were performed with *n* = 5 mice per group per time point. Cell-based experiments were repeated at least three independent times, each with technical triplicates. Normality of data distribution was assessed using Shapiro–Wilk test, and homogeneity of variances was confirmed by Levene’s test. For comparisons between two groups, unpaired two-tailed Student’s *t*-test was used. For comparisons among multiple groups, one-way analysis of variance (ANOVA) followed by Tukey’s multiple comparison test was applied. For comparisons involving two factors (e.g., time and treatment), two-way ANOVA with Tukey’s post hoc test was used. Statistical analyses were performed using SPSS (version 23.0, SPSS Inc., Chicago, IL, USA) and GraphPad Prism (version 8.0, GraphPad Software, La Jolla, CA, USA). Differences were considered statistically significant at * *p* < 0.05, ** *p* < 0.01, and *** *p* < 0.001. Non-significant differences are denoted as “ns”.

## 3. Results

### 3.1. Transcriptional Regulation of the PI3K/Akt/mTOR Autophagy Pathway by E. coli HPI in the Mouse Intestine

**Figure 1:**
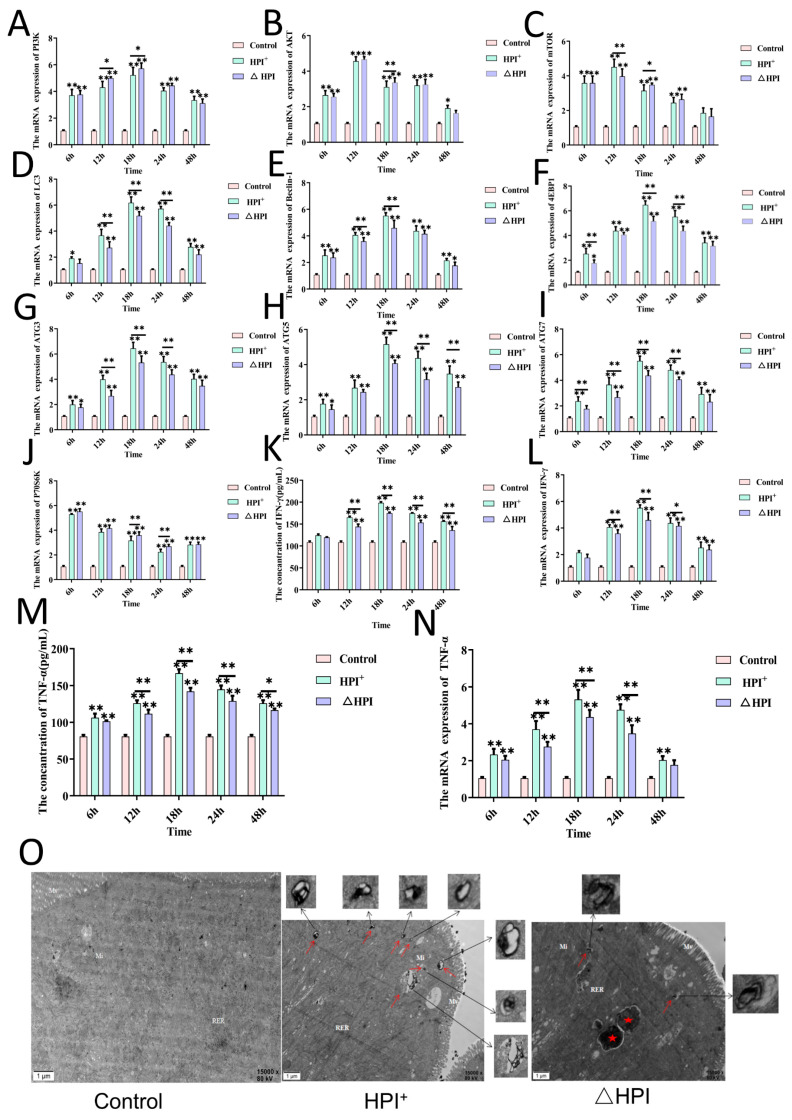
Transcriptional regulation of the PI3K/Akt/mTOR autophagy pathway by *E. coli* HPI in the mouse duodenum. (**A**–**J**) Relative mRNA expression of autophagy-related genes, including the upstream negative regulators (*PI3K*, *Akt*, and *mTOR*) and execution-related genes (*Beclin-1*, *LC3*, *4EBP1*, *ATG3*, *ATG5*, *ATG7*, and *p70S6K*), in mouse duodenum at indicated time points post-infection, measured by RT-qPCR (*n* = 3 per group). (**K**,**L**) Protein (ELISA) and mRNA (RT-qPCR) levels of *IFN-γ* in mouse duodenum (*n* = 3 per group). (**M**,**N**) Protein (ELISA) and mRNA (RT-qPCR) levels of *TNF-α* in mouse duodenum (*n* = 3 per group). (**O**) TEM images of duodenal epithelial cells at 18 h post-infection (15,000×). Red arrows indicate autophagosomes; five-pointed stars indicate autophagosomes containing organelles and cytoplasmic contents. Data are presented as mean ± SD. ** *p* < 0.01, * *p* < 0.05. ΔHPI: HPI-deficient mutant.

**Figure A1:**
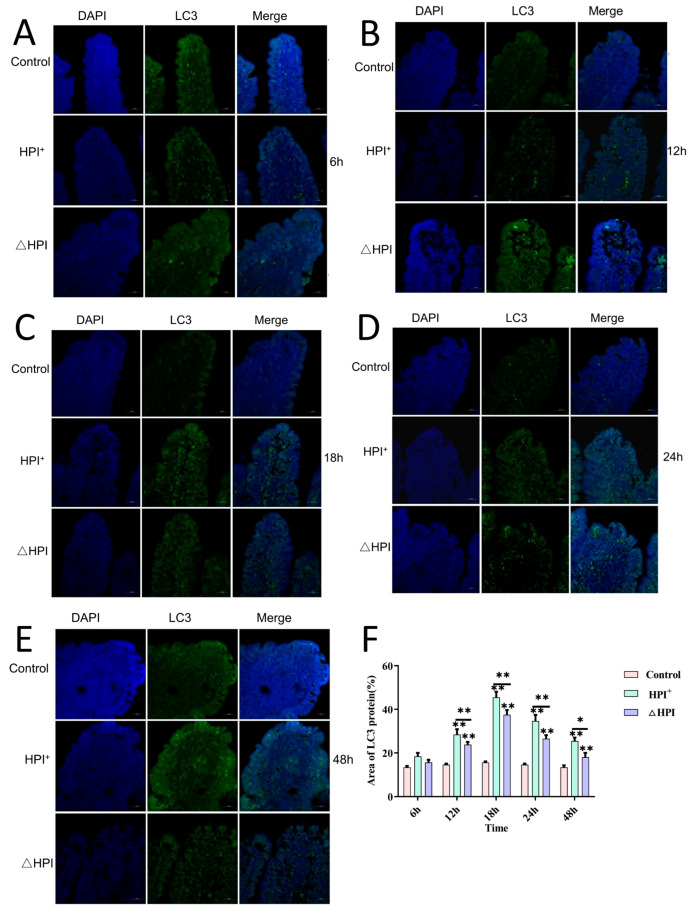
Immunofluorescence localization and quantification of LC3 in mouse duodenum. (**A**–**E**) Representative immunofluorescence images of LC3 (green) in mouse duodenum at 6, 12, 18, 24, and 48 h post-infection (*n* = 5 mice per group). Nuclei were counterstained with DAPI (blue). Scale bar = 20 μm. (**F**) Quantification of LC3 immunofluorescence intensity in mouse duodenum at 6, 12, 18, 24, and 48 h post-infection (*n* = 5 mice per group). Data are presented as mean ± SD. ** *p* < 0.01, * *p* < 0.05.

Given the established role of bacterial pathogens in modulating host autophagy, we examined whether *E. coli* HPI influences the transcriptional regulation of the PI3K/Akt/mTOR pathway. RT-qPCR analysis revealed that *E. coli* HPI^+^ infection reprogrammed the host autophagic response, significantly downregulating key upstream inhibitors of autophagy (*PI3K*, *Akt*, and *mTOR*) while concurrently enhancing the expression of autophagy execution genes (*Beclin-1* and *LC3*). Similar expression patterns were observed for other autophagy-related genes, including *p70S6 kinase (p70S6K)*, *autophagy-related gene 3 (ATG3)*, *autophagy-related gene 5 (ATG5)*, *autophagy-related gene 7 (ATG7)* and *eukaryotic translation initiation factor 4e-binding protein 1 (4EBP1)* (Figure 1A,J). We next examined whether *E. coli* HPI-mediated autophagy influenced inflammatory cytokine production. Both ELISA and RT-qPCR showed that *E. coli* infection triggered a transient increase in TNF-α and IFN-γ, peaking at 18 h. This inflammatory response was significantly amplified by HPI, with cytokine levels higher in *E. coli* HPI^+^-infected mice from 12 to 48 h (Figure 1K,N). To spatially localize autophagic activity in intestinal tissues, we performed immunofluorescence staining for LC3 in duodenal sections from control, *E. coli* HPI^+^, and *E. coli* ΔHPI-infected mice. As shown in Figure A1, LC3-positive signals (green fluorescence) were predominantly distributed in the duodenal epithelium and lamina propria, with minimal staining observed in uninfected controls. Quantitative analysis of LC3 fluorescence intensity across multiple time points (6, 12, 18, 24, and 48 h post-infection) revealed a time-dependent biphasic pattern: fluorescence intensity progressively increased from 6 h, peaked at 18 h, and subsequently declined thereafter. Notably, the *E. coli* HPI^+^ group consistently exhibited higher LC3 fluorescence intensity than the ΔHPI group at all measured time points, with statistically significant differences observed at 12, 18, 24, and 48 h. These findings indicate that HPI upregulates *E. coli*-induced LC3 expression in the mouse duodenum and that autophagic activity in intestinal tissues reaches its maximum at 18 h post-infection. To complement the above findings with ultrastructural evidence, we performed TEM at 18 h post-infection. This revealed that *E. coli* HPI^+^ infection exacerbated epithelial damage, including blunted microvilli and swollen organelles. Furthermore, duodenal cells from *E. coli* HPI^+^-infected mice contained a greater number of autophagosomes, suggesting that HPI not only aggravates pathology but also potently enhances the autophagic response to infection (Figure 1O). These results suggest that HPI^+^ infection induced both autophagy and more severe pathology. To determine whether the induced autophagy is protective or detrimental, we next employed pharmacological modulators (rapamycin and 3-MA) in macrophage infection models.

### 3.2. Transcriptional Regulation of the PI3K/Akt/mTOR Autophagy Pathway by Autophagy Modulators During E. coli HPI Infection

**Figure 2:**
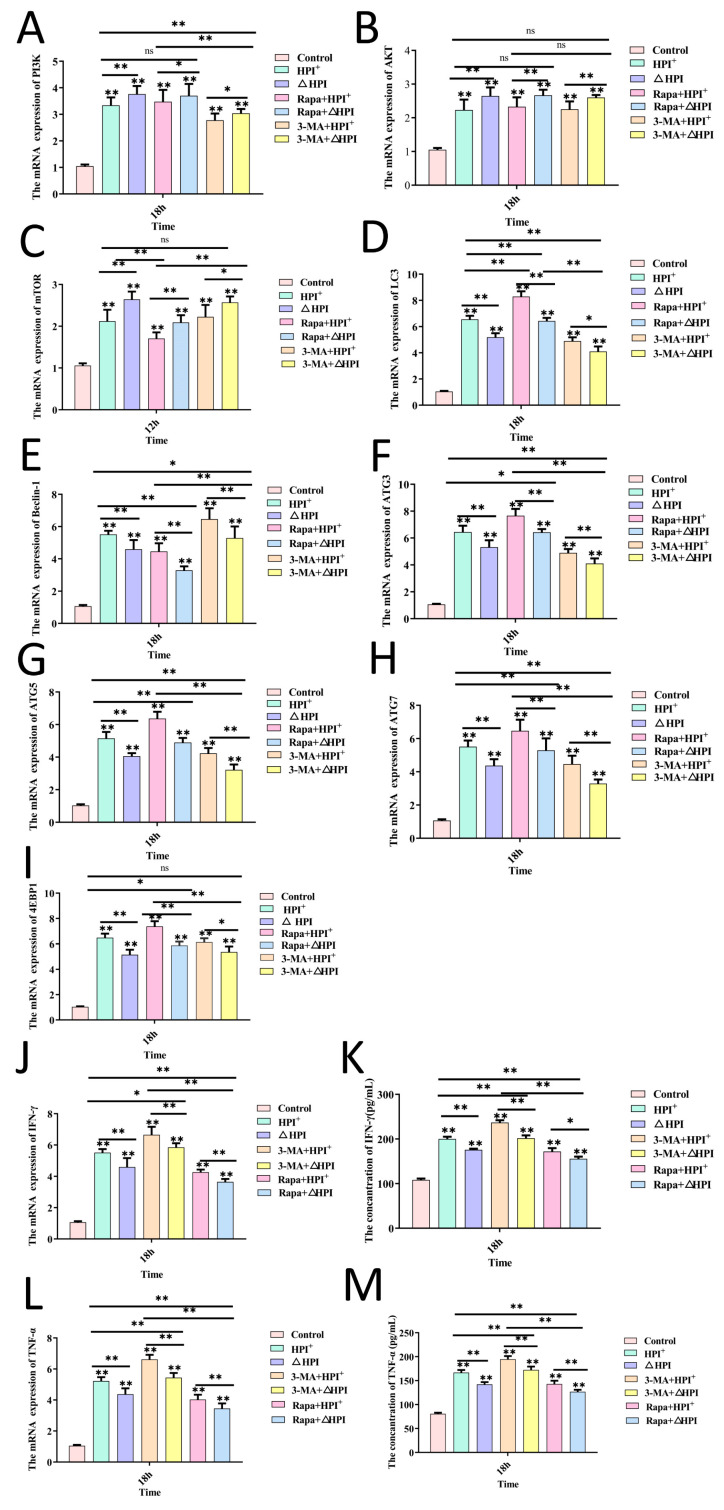
Transcriptional regulation of the PI3K/Akt/mTOR autophagy pathway by pharmacological modulators during *E. coli* HPI infection. (**A**–**I**) Relative mRNA expression of autophagy-related genes, including the upstream negative regulators (*PI3K*, *Akt*, and *mTOR*) and execution-related genes (*Beclin-1*, *LC3*, *ATG3*, *ATG5*, *ATG7*, and *4EBP1*) in the PI3K/Akt/mTOR autophagic signaling pathway in mouse intestinal tissues, measured by RT-qPCR (*n* = 3 per group). (**J**,**K**) Protein (ELISA) and mRNA (RT-qPCR) levels of *IFN-γ* in mouse duodenum (*n* = 5 mice per group). (**L**,**M**) Protein (ELISA) and mRNA (RT-qPCR) levels of *TNF-α* in mouse duodenum (*n* = 5 mice per group). Data are presented as mean ± SD. ** *p* < 0.01, * *p* < 0.05; ns, not significant (*p* > 0.05). ΔHPI: HPI-deficient mutant.

**Figure A2:**
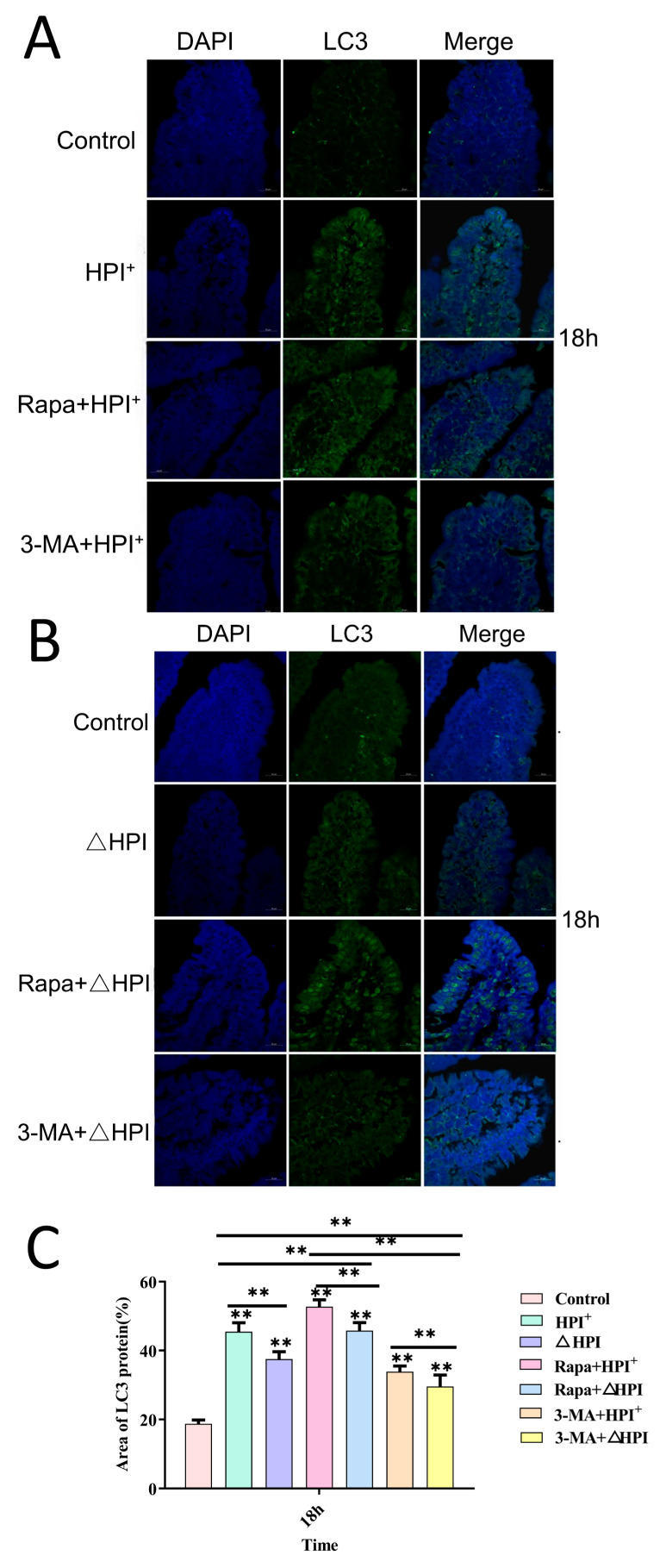
Immunofluorescence localization and quantification of LC3 in mouse duodenum following pharmacological modulation of autophagy. (**A**,**B**) Representative immunofluorescence images of LC3 (green) in mouse duodenum. Nuclei were counterstained with DAPI (blue). Scale bar = 20 μm. (**C**) Quantification of LC3 immunofluorescence intensity (*n* = 5 mice per group). ** *p* < 0.01.

**Figure A3:**
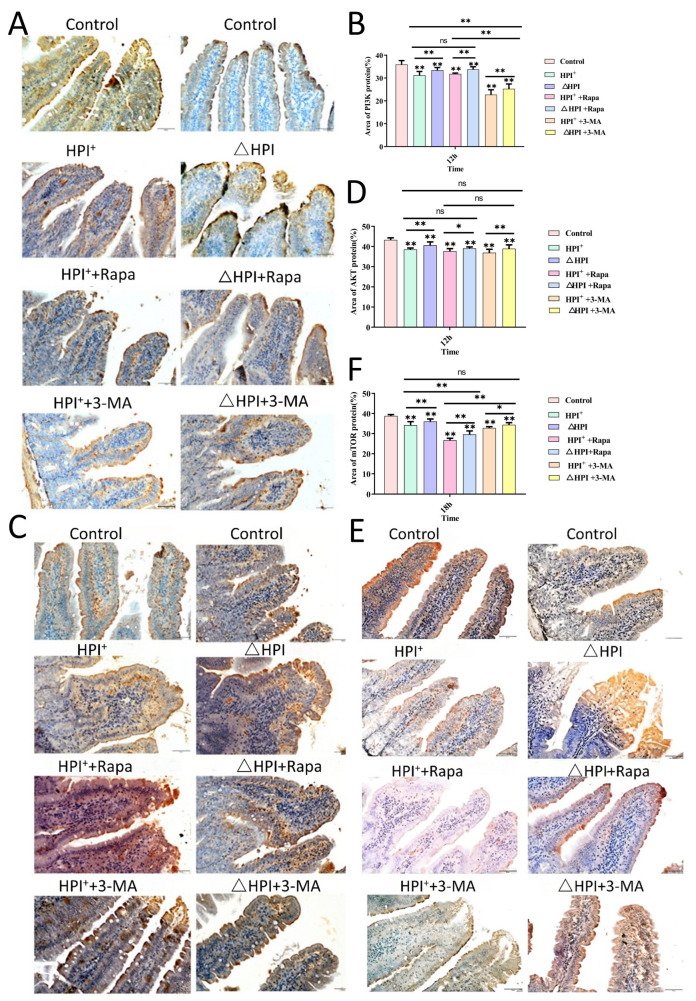
Immunohistochemical localization and quantification of PI3K, Akt, and mTOR in mouse duodenum following pharmacological modulation of autophagy. (**A**) Representative immunohistochemical images of PI3K in mouse duodenum (200×). (**B**) Quantification of PI3K immunohistochemical staining (*n* = 5 mice per group). (**C**) Representative immunohistochemical images of Akt in mouse duodenum (200×). (**D**) Quantification of Akt immunohistochemical staining (*n* = 5 mice per group). (**E**) Representative immunohistochemical images of mTOR in mouse duodenum (200×). (**F**) Quantification of mTOR immunohistochemical staining (*n* = 5 mice per group). Data are presented as mean ± SD. ** *p* < 0.01, * *p* < 0.05; ns, not significant.

To delineate the functional interplay between the HPI and the autophagy pathway, we employed pharmacological modulators—rapamycin as an inducer and 3-MA as an inhibitor—in conjunction with *E. coli* HPI^+^ and ΔHPI infection. We first examined the transcriptional responses to pharmacological modulation. RT-qPCR revealed that both rapamycin and 3-MA differentially regulated autophagy-related genes, with HPI consistently reinforcing a pro-autophagic profile. Rapamycin significantly downregulated *mTOR* and *Beclin-1*, while upregulating autophagy execution genes (*ATG3*, *ATG5*, *ATG7* and *LC3*) as well as *4EBP1*, without affecting *PI3K* or *Akt*. In contrast, 3-MA suppressed *PI3K* and the same suite of execution genes, but did not alter *Akt* or *mTOR* expression, and significantly elevated *Beclin-1* (Figure 2A,I). We then asked whether autophagy modulation influenced inflammatory outcomes. RT-qPCR and ELISA analyses showed that rapamycin-induced autophagy significantly suppressed both mRNA and protein levels of *TNF-α* and *IFN-γ*, whereas 3-MA-mediated autophagy inhibition enhanced their expression. Strikingly, rapamycin induced stronger suppression in the ΔHPI + Rapa group, while 3-MA induced stronger inflammation in the HPI^+^ + 3-MA group (Figure 2J,M). To spatially validate these transcriptional changes, we performed immunofluorescence staining for LC3 in intestinal tissues. Rapamycin enhanced LC3 fluorescence, indicative of elevated autophagic activity, and this effect was more pronounced in the HPI^+^ + Rapa group. In contrast, 3-MA suppressed LC3 signal, with the weakest intensity observed in the ΔHPI + 3-MA group, consistent with its role as an autophagy inhibitor (Figure A2). To localize and semi-quantify these changes at the tissue level, we examined the protein expression of PI3K, Akt, and mTOR in mouse intestine by immunohistochemistry following treatment with 3-MA or rapamycin, combined with *E. coli* HPI^+^ or ΔHPI infection (Figure A3). 3-MA treatment significantly reduced PI3K expression in both HPI^+^ and ΔHPI backgrounds compared to infection alone or rapamycin co-treatment, indicating effective suppression of HPI-induced PI3K expression, whereas rapamycin had no effect on PI3K. Akt expression was unaffected by either 3-MA or rapamycin. In contrast, rapamycin dramatically suppressed mTOR expression in both infection backgrounds, confirming its inhibitory effect on mTOR. Notably, across all treatment conditions, the HPI^+^ groups consistently exhibited lower expression of PI3K, Akt, and mTOR than their corresponding ΔHPI groups, further demonstrating that HPI exerts a negative regulatory role on the PI3K/Akt/mTOR axis during *E. coli* infection.

### 3.3. E. coli HPI Promotes M Cells Differentiation and Mucosal Immune Responses in the Mouse Intestine System

**Figure 3:**
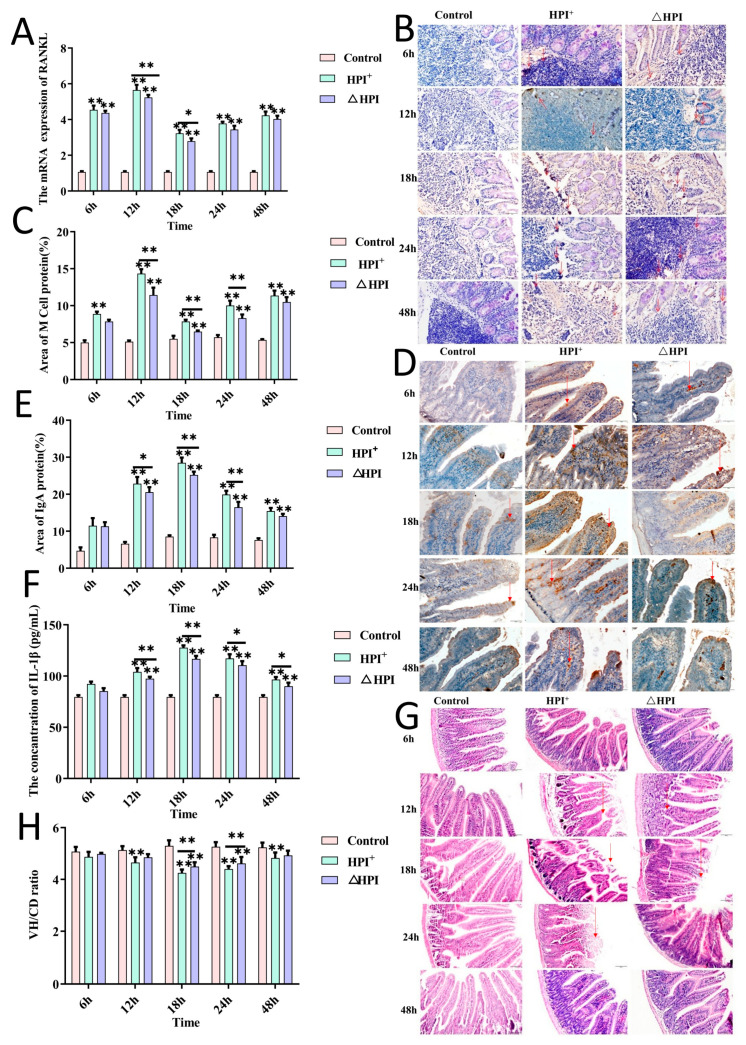
Effects of *E. coli* HPI on M cells in the intestinal mucosa of mice. (**A**) Relative mRNA expression of *RANKL*, a key regulator of intestinal M cells differentiation, measured by RT-qPCR (*n* = 5 mice per group). (**B**) Representative immunohistochemical images of M cells in mouse duodenum (200×). Red arrows indicate positive M-cell staining (brown). (**C**) Quantification of M-cell immunohistochemical staining (*n* = 5 mice per group). (**D**) Representative immunohistochemical images of IgA in mouse duodenum (200×). Red arrows indicate positive IgA staining (brownish-yellow). (**E**) Quantification of IgA immunohistochemical staining (*n* = 5 mice per group). (**F**) Protein levels of IL-1β in mouse duodenum measured by ELISA (*n* = 5 mice per group). (**G**) Representative H&E-stained sections of mouse duodenum (200×). Red arrows indicate villus shortening, disintegration, and loss of mucosal integrity. (**H**) Villus-to-crypt ratio in mouse duodenum (*n* = 5 mice per group). Data are presented as mean ± SD. ** *p* < 0.01, * *p* < 0.05. ΔHPI: HPI-deficient mutant.

**Figure A4:**
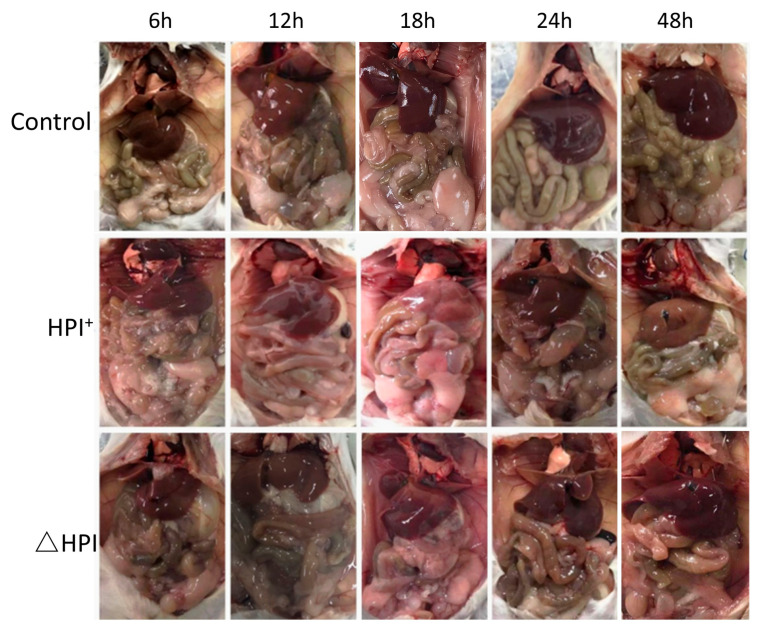
Changes in necropsy in mice under different infection conditions at 6, 12, 18, 24, and 48 h post-infection.

To comprehensively evaluate the impact of HPI on intestinal pathology and mucosal immunity, we performed a series of gross, histological, and immunological examinations. To gain an initial overview of HPI-induced pathogenesis, we performed gross necropsy examinations at 6, 12, 18, 24, and 48 h post-infection (Figure A4). In the control group, the intestinal contents were abundant, and the heart, liver, spleen, lungs, and kidneys appeared normal in size and morphology with no visible lesions. In contrast, the experimental groups exhibited marked pathological changes: the intestinal tract was distended with thin, yellowish, and foul-smelling contents; the intestinal wall was translucent and thinned; the mucosa appeared hyperemic with occasional petechial hemorrhages; and the liver and spleen showed varying degrees of hemorrhage. Notably, the gross lesions in the *E. coli* ΔHPI group were consistently milder than those in the *E. coli* HPI^+^ group across all time points, suggesting that HPI exacerbates systemic and intestinal pathology. Given that HPI modulates both intracellular autophagic responses and mucosal immune homeostasis, we next examined its effect on M cells, the key antigen-sampling cells that initiate mucosal immunity. To this end, we assessed the impact of *E. coli* HPI^+^ and ΔHPI on key markers of M-cell biology and mucosal immunity. Infection with *E. coli* significantly and in a time-dependent manner upregulated mRNA expression of *RANKL*, a principal mediator of intestinal M-cell differentiation. Importantly, this induction was markedly potentiated by the presence of HPI, with RANKL levels in *E. coli* HPI^+^-infected mice substantially exceeding those in the *E. coli* ΔHPI group at 12 and 18 h post-infection (Figure 3A). Consistent with this transcriptional profile, immunohistochemical analysis revealed a transient infection-induced increase in M-cell numbers, which was again significantly amplified by HPI at 12, 18, and 24 h (Figure 3B,C). We next asked whether HPI-enhanced M-cell differentiation influenced mucosal immunoglobulin responses. Corroborating the M-cell data, *E. coli* infection triggered a transient rise in duodenal IgA, peaking at 18 h. Remarkably, the presence of *E. coli* HPI robustly enhanced this IgA response at 12, 18, and 24 h (Figure 3D,E). Given the established link between innate immunity and M-cell regulation, we profiled the pro-inflammatory cytokine IL-1β. ELISA results confirmed that intestinal IL-1β levels were elevated post-infection and, critically, were consistently higher in *E. coli* HPI^+^-infected mice compared to the *E. coli* ΔHPI group, underscoring a role of HPI in potentiating this inflammatory response (Figure 3F). To determine whether the above immunological and autophagic alterations ultimately translated into demonstrable tissue injury, we evaluated intestinal histopathology by H&E staining. H&E showed that infection induced damage in a time-dependent manner in the duodenum, characterized by villus shortening, disintegration, and epithelial exfoliation. Quantitative analysis of the villus-to-crypt ratio confirmed the most severe injury at 18 h. Strikingly, this structural damage was significantly exacerbated by HPI, as reflected by a markedly lower ratio in *E. coli* HPI^+^-infected mice at 18 and 24 h compared to the *E. coli* ΔHPI (Figure 3G,H).

### 3.4. E. coli HPI Enhances Autophagic Activation in Macrophages

**Figure 4:**
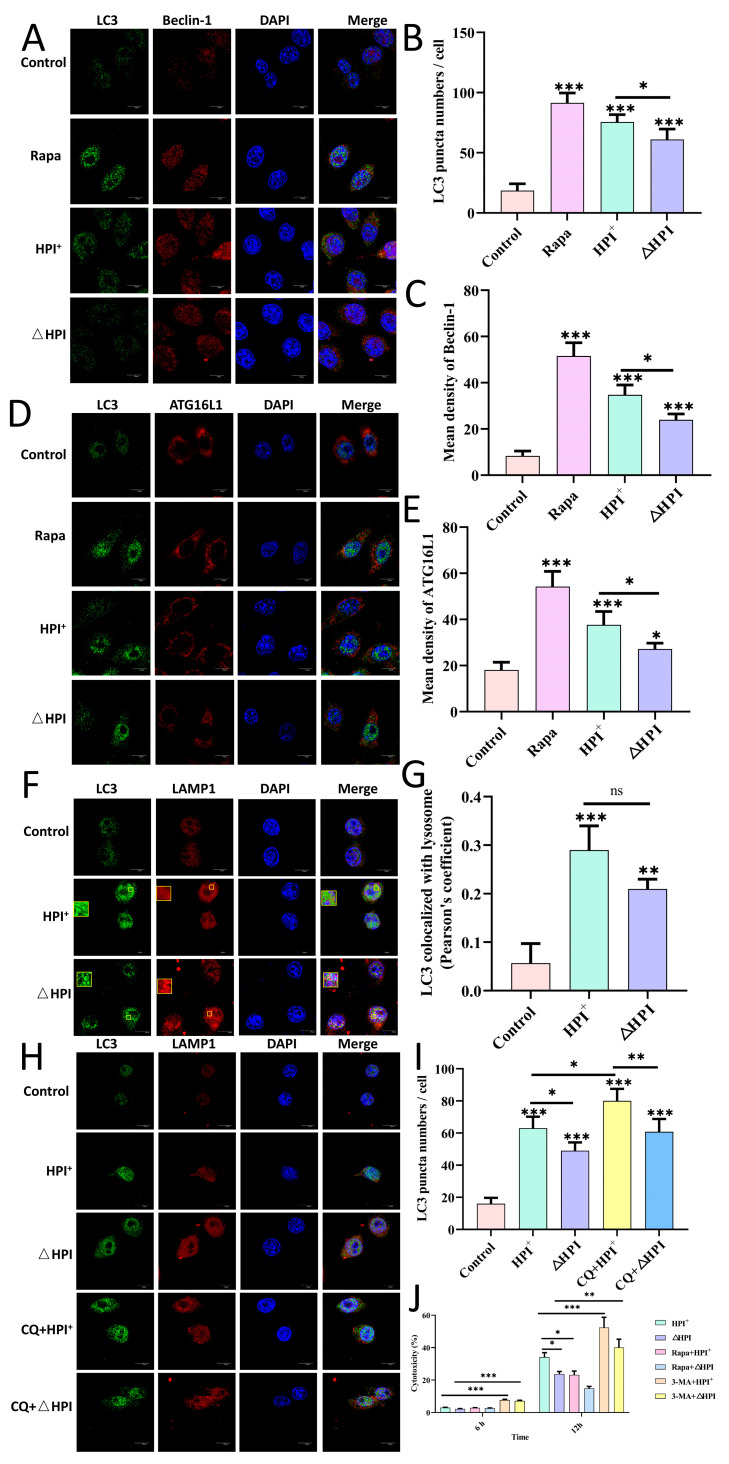
*E. coli* HPI enhances autophagic activation in RAW264.7 macrophages. (**A**) Representative confocal microscopy images of LC3 (green) and Beclin-1 (red) in RAW264.7 cells. Nuclei were counterstained with DAPI (blue). Scale bar = 10 μm (*n* = 3 independent experiments). (**B**) Quantification of LC3 fluorescence intensity (*n* = 3 independent experiments). (**C**) Quantification of Beclin-1 fluorescence intensity (*n* = 3 independent experiments). (**D**) Representative confocal images of LC3 (green) and ATG16L1 (red) in RAW264.7 cells. Scale bar = 10 μm. (**E**) Quantification of LC3 and ATG16L1 fluorescence intensity (*n* = 3 independent experiments). (**F**) Representative confocal images of LC3 (green) and LAMP1 (red) in RAW264.7 cells. Enlarged views of individual cells are shown in the insets for HPI^+^ and ΔHPI groups. Scale bar = 10 μm. (**G**) Quantification of LC3 and LAMP1 colocalization (Pearson’s correlation coefficient) (*n* = 3 independent experiments). (**H**) Effect of chloroquine (CQ) on LC3 (green) and LAMP1 (red) distribution in RAW264.7 cells. Scale bar = 10 μm. (**I**) Quantification of LC3 puncta per cell after CQ treatment (*n* = 3 independent experiments). (**J**) LDH release in RAW264.7 cells pretreated with rapamycin or 3-MA followed by *E. coli* infection at 6 and 12 h post-infection (*n* = 3 independent experiments). Data are presented as mean ± SD. *** *p* < 0.001, ** *p* < 0.01, * *p* < 0.05; ns, not significant (*p* > 0.05). ΔHPI: HPI-deficient mutant.

Having observed HPI-mediated autophagic activation in intestinal tissues, we next asked whether a similar response occurs in macrophages, a primary target of bacterial infection and a key cell type for autophagic regulation. Immunofluorescence analysis provided further spatial resolution of autophagic activation. Both strains induced the formation of distinct LC3-positive puncta at 6 h post-infection, suggesting autophagosome accumulation. Quantitative analysis showed significantly higher LC3 puncta in *E. coli* HPI^+^-infected cells compared to the ΔHPI group. Consistently, Beclin-1 expression was markedly upregulated in infected groups, with the HPI^+^ group exhibiting stronger intensity than the ΔHPI group, suggesting that HPI enhances autophagic vesicle formation and upregulates core autophagic proteins (Figure 4A,C). We next examined whether the enhanced LC3 puncta formation was associated with increased expression of Autophagy-related 16-like 1 (ATG16L1), a core component of the autophagy-related protein complex required for autophagosome formation. Immunofluorescence analysis revealed that ATG16L1 was significantly upregulated following rapamycin treatment or infection with either strain. The increase in ATG16L1 was more pronounced in *E. coli* HPI^+^-infected cells than in the ΔHPI group. Double staining further revealed partial colocalization of LC3 and ATG16L1 (Figure 4D,E). To determine whether autophagosomes matured properly, we assessed autophagosome–lysosome fusion. Immunofluorescence analysis revealed increased co-localization of LC3 with the lysosomal marker Lysosome-Associated Membrane Protein 1 (LAMP1) in both infected groups, suggesting enhanced autophagosome-lysosome fusion. Quantitative analysis using Pearson’s correlation coefficient showed a higher mean value in *E. coli* HPI^+^-infected cells (0.29) compared to the ΔHPI group (0.21), although this difference was not statistically significant (Figure 4F,G). We then assessed autophagic flux using CQ, which inhibits lysosomal degradation. CQ pretreatment resulted in significantly more LC3 puncta in *E. coli* HPI^+^-infected cells compared to *E. coli* ΔHPI-infected cells, consistent with enhanced autophagic flux in the HPI^+^ group, whereas the ΔHPI group showed only a slight, non-significant increase upon CQ treatment (Figure 4H,I). Finally, to assess the functional consequences of autophagy modulation in macrophage viability, we measured LDH release. RAW264.7 cells were pretreated with rapamycin or 3-MA and infected with *E. coli* strains, with LDH release measured at 6 and 12 h. At 6 h, LDH release remained low (<5%) in all groups except 3-MA-pretreated cells, which showed elevated levels. By 12 h, *E. coli* HPI^+^ infection resulted in significantly higher LDH release than *E. coli* ΔHPI infection. Rapamycin significantly reduced LDH release in *E. coli* HPI^+^-infected cells, whereas 3-MA further elevated LDH levels in both infected groups (Figure 4J).

### 3.5. Transcriptional Regulation of Autophagy-Related Genes by E. coli HPI in Macrophages

**Figure 5:**
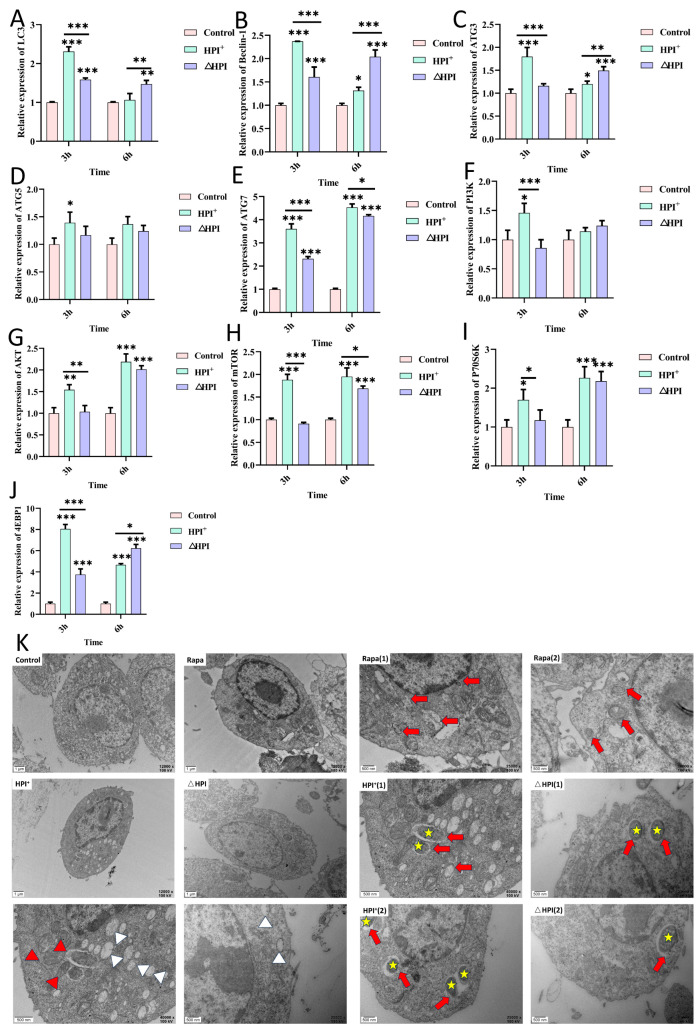
Transcriptional dynamics of the PI3K/Akt/mTOR pathway in *E. coli* HPI-infected macrophages. (**A**–**J**) Relative mRNA expression of autophagy-related genes, including the upstream negative regulators (*PI3K*, *Akt*, and *mTOR*) and execution-related genes (*LC3*, *Beclin-1*, *ATG3*, *ATG5*, *ATG7*, *p70S6K*, and *4EBP1*), in RAW264.7 cells infected with *E. coli* HPI^+^ or ΔHPI at 3 and 6 h post-infection (*n* = 3 independent experiments). (**K**) TEM images of RAW264.7 cells under indicated conditions (12,000×). Red arrows indicate autophagic vesicles, yellow stars indicate intracellular bacteria, red triangles indicate aberrant mitochondria, and white triangles indicate vacuolated mitochondria. Scale bar = 1 μm (*n* = 3 independent experiments). Data are presented as mean ± SD. *** *p* < 0.001, ** *p* < 0.01, * *p* < 0.05.

To further verify whether autophagy was indeed activated under these conditions, we examined the mRNA expression of autophagy-related genes by RT-qPCR and directly visualized autophagosome formation by TEM. RT-qPCR analysis revealed that *E. coli* HPI^+^ infection induced a more rapid and pronounced upregulation of autophagy-related genes compared to the *E. coli* ΔHPI strain. At 3 h post-infection, *E. coli* HPI^+^ infection resulted in significantly higher expression of *LC3*, *Beclin-1*, *ATG3*, and *ATG7* relative to the ΔHPI group. By 6 h, this expression pattern shifted, with *E. coli* ΔHPI-infected cells showing higher levels of *LC3*, *Beclin-1*, and *ATG3*. *ATG5* expression was significantly upregulated in the HPI^+^ group at 3 h post-infection, while *ATG7* expression remained significantly elevated in the HPI^+^ group at both time points. For the PI3K/Akt/mTOR axis, *E. coli* HPI^+^ infection accelerated the transcriptional activation of this pathway relative to the ΔHPI strain. *PI3K* expression was significantly elevated at 3 h in the HPI^+^ group. Consistently, *Akt*, *mTOR*, and *p70S6K* showed earlier upregulation at 3 h in *E. coli* HPI^+^-infected cells, whereas their induction in the ΔHPI group was delayed until 6 h. Although *4EBP1* was strongly induced at 3 h in both groups, its expression declined more rapidly in the *E. coli* HPI^+^ group, falling slightly below the *E. coli* ΔHPI level by 6 h (Figure 5A,J). To visualize the ultrastructural correlates of these transcriptional changes, we also performed TEM at 6 h post-infection. Cells infected with either strain maintained intact cellular architecture, characterized by distinct nuclear membranes, prominent nucleoli, and normal chromatin distribution. However, *E. coli* HPI^+^-infected cells exhibited prominent cytoplasmic vacuolization. Intracellular bacteria were observed in both groups, with bacteria enclosed within autophagic vesicles in each; notably, the HPI^+^ group contained more bacteria and associated autophagic vesicles than the ΔHPI group. Cells treated with rapamycin displayed two types of autophagic vesicle structures: autophagosomes (double-membrane) and autolysosomes (single-membrane), as indicated by red arrows. Furthermore, mitochondrial damage was evident in both infected groups, manifesting as mitochondrial swelling, disintegrated and vacuolated cristae, and the appearance of dumbbell-shaped aberrant mitochondria. Red triangles indicate aberrant mitochondria (including dumbbell-shaped and swollen forms), and white triangles indicate vacuolated mitochondria resulting from cristae loss and content dissolution. The *E. coli* HPI^+^ group exhibited more severe mitochondrial damage, including a greater abundance of vacuolated mitochondria. Collectively, these ultrastructural observations suggest that *E. coli* HPI^+^ infection induces the formation of autophagic vesicles and exacerbates mitochondrial damage in RAW264.7 cells, providing morphological evidence that HPI enhances bacterial-induced autophagy and cellular injury (Figure 5K).

### 3.6. Regulation of Inflammatory Responses and Cell Survival by Autophagy in E. coli HPI Infection

**Figure 6:**
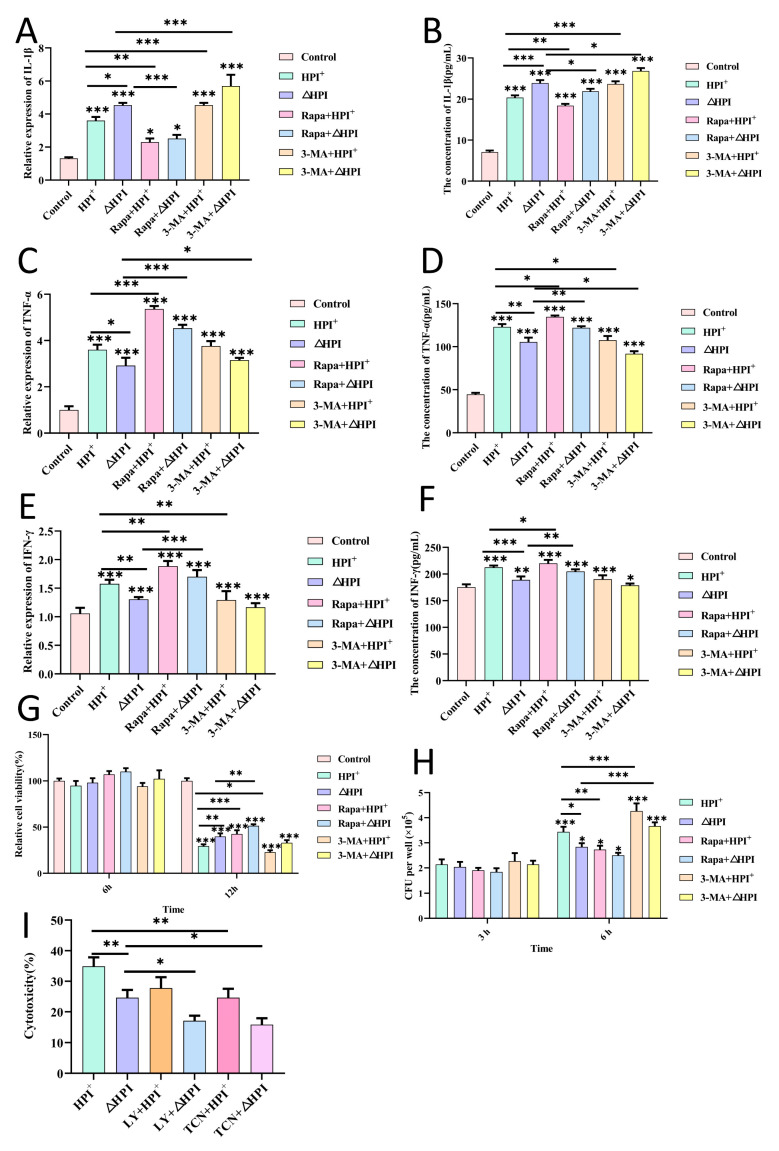
Effects of Autophagy on Inflammatory Responses and Cell Viability in *E. coli* HPI Infection. (**A**,**B**) mRNA (by RT-qPCR) and protein (by ELISA) levels of *IL-1β* in RAW264.7 cells following treatment with rapamycin or 3-MA combined with *E. coli* HPI^+^ or ΔHPI infection (*n* = 3 per group). (**C**,**D**) mRNA (by RT-qPCR) and protein (by ELISA) levels of *TNF-α* in RAW264.7 cells following treatment with rapamycin or 3-MA combined with *E. coli* HPI^+^ or ΔHPI infection (*n* = 3 per group). (**E**,**F**) mRNA (by RT-qPCR) and protein (by ELISA) levels of *IFN-γ* in RAW264.7 cells following treatment with rapamycin or 3-MA combined with *E. coli* HPI^+^ or ΔHPI infection (*n* = 3 per group). (**G**) Cell viability of RAW264.7 cells measured by CCK-8 assay at 6 and 12 h post-infection following treatment with rapamycin or 3-MA combined with *E. coli* HPI^+^ or ΔHPI infection (*n* = 3 per group). (**H**) Intracellular bacterial loads in RAW264.7 cells at 3 and 6 h post-infection following treatment with rapamycin or 3-MA combined with *E. coli* HPI^+^ or ΔHPI infection (*n* = 3 per group). (**I**) Effects of LY and TCN pretreatment on RAW264.7 cell damage after *E. coli* HPI infection, measured by LDH release (*n* = 3 independent experiments). *** *p* < 0.001, ** *p* < 0.01, * *p* < 0.05.

To investigate the functional interplay between autophagy and host defense during *E. coli* HPI^+^ infection, we assessed cytokine profiles, cell viability, and bacterial survival under modulated autophagic conditions. Rapamycin-induced autophagy significantly suppressed *IL-1β* expression at both mRNA (by RT-qPCR) and protein (by ELISA) levels, whereas 3-MA-mediated autophagy inhibition further enhanced IL-1β production. In contrast, *TNF-α* and *IFN-γ* levels were elevated by rapamycin but diminished by 3-MA. *E. coli* HPI^+^ infection led to reduced *IL-1β* but increased *TNF-α* and *IFN-γ* compared to the *E. coli* ΔHPI strain, suggesting that *E. coli* HPI alters the cytokine landscape in an autophagy-dependent manner (Figure 6A,F). We further examined whether autophagy influenced host cell viability in response to infection. CCK-8 assays revealed that although viability remained largely unaffected at 6 h post-infection, a significant decrease occurred by 12 h. The *E. coli* HPI^+^ strain induced more severe cytotoxicity than the *E. coli* ΔHPI strain. Rapamycin increased cell viability in both infection contexts, whereas 3-MA further compromised cell survival in *E. coli* HPI^+^-infected macrophages (Figure 6G). To determine whether autophagic activity affects intracellular bacteria, we quantified bacterial loads. While bacterial loads were comparable across all groups at 3 h, a marked divergence emerged by 6 h, with *E. coli* HPI^+^ strains exhibiting enhanced survival over ΔHPI strains. Rapamycin reduced the intracellular burden of *E. coli* HPI^+^, and conversely, 3-MA enhanced it (Figure 6H). These results suggest that HPI augments intramacrophage survival and that autophagy activation restricts *E. coli* persistence, whereas autophagy inhibition favors bacterial proliferation. Subsequently, to evaluate the functional relevance of autophagy modulation in infection outcome, we measured LDH release in cells pretreated with TCN or LY. TCN significantly attenuated cellular damage induced by both bacterial strains, indicating a broad protective effect. LY pretreatment significantly reduced LDH release in *E. coli* ΔHPI-infected cells, while in HPI^+^-infected cells, a similar trend was observed but did not reach statistical significance (Figure 6I). These results suggest that pharmacological modulation of autophagy can mitigate infection-associated cytotoxicity, although the extent of protection may vary depending on the bacterial strain and the modulator.

### 3.7. Beclin-1 Knockdown Attenuates HPI-Induced Autophagy in Macrophages

**Figure 7:**
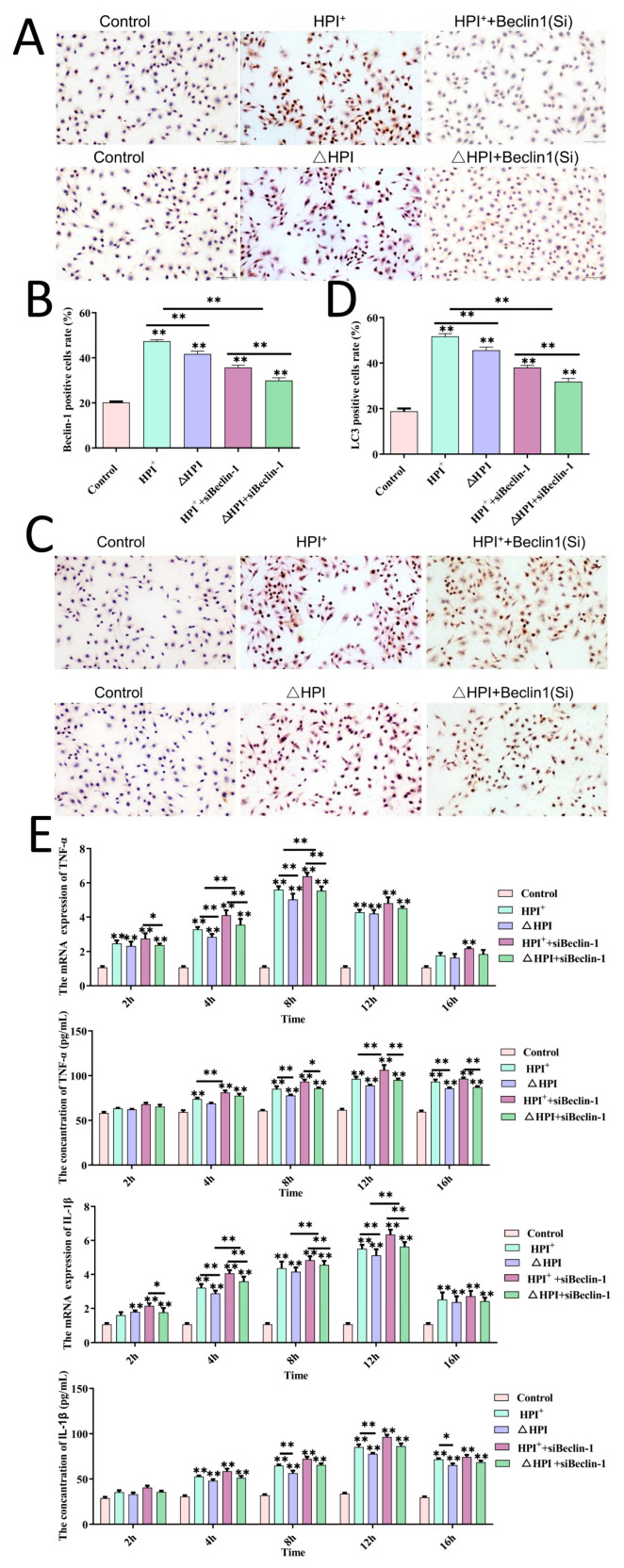
Beclin-1 knockdown suppresses autophagy and exacerbates inflammation. (**A**) Immunohistochemical results of *Beclin-1* in RAW264.7 cells (400×). (**B**) Quantification of *Beclin-1* immunohistochemistry intensity (*n* = 3 per group). (**C**) *LC3* immunohistochemistry results in RAW264.7 cells (400×). (**D**) Quantification of *LC3* immunohistochemistry intensity (*n* = 3 per group). (**E**) mRNA (by RT-qPCR) and protein (by ELISA) levels of *IL-1β* and *TNF-α* in RAW264.7 cells following *Beclin-1* knockdown and *E. coli* infection (*n* = 3 independent experiments). Data are presented as mean ± SD. ** *p* < 0.01, * *p* < 0.05; ΔHPI: HPI-deficient mutant.

**Figure A5:**
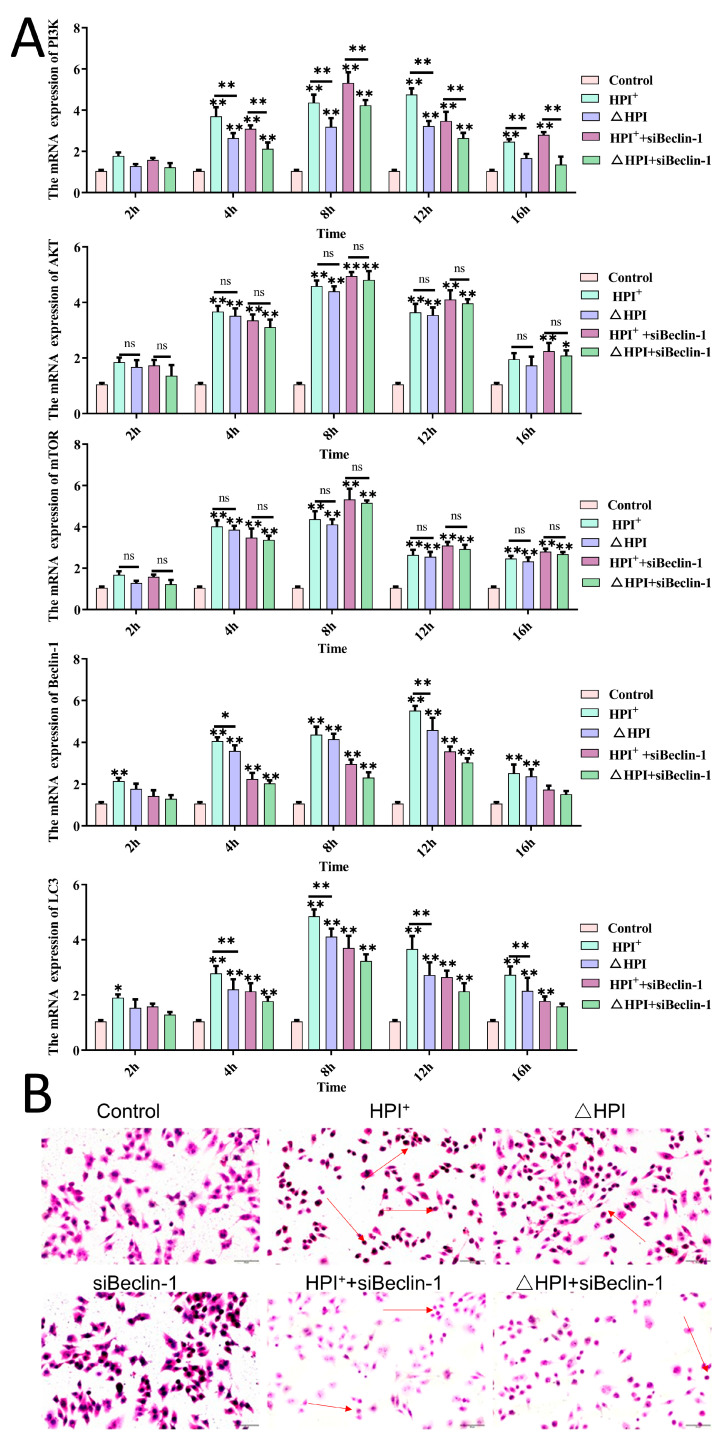
Transcriptional changes and morphological alterations upon Beclin-1 knockdown in HPI-infected macrophages. (**A**) Relative mRNA expression of mTOR, PI3K, Akt, Beclin-1, and LC3 in RAW264.7 cells measured by RT-qPCR (*n* = 3 per group). (**B**) Representative H&E-stained images of RAW264.7 cells (400×). Red arrows indicate cells with rounded or oval morphology and swelling, indicative of cellular injury or activation. Data are presented as mean ± SD. ** *p* < 0.01, * *p* < 0.05; ns, not significant.

To genetically validate the functional role of *Beclin-1* in *E. coli* HPI-mediated autophagy, we first established a *Beclin-1* knockdown model in RAW264.7 cells. We next examined the transcriptional dynamics of autophagy-related genes in this context. While *E. coli* HPI^+^ infection time-dependently upregulated multiple autophagy-related genes, including the negative regulators *PI3K*, *Akt*, *mTOR*, and the execution-related genes *Beclin-1* and *LC3* in control siRNA-transfected cells, *Beclin-1* knockdown significantly blunted the induction of *PI3K*, *Beclin-1*, and *LC3* in response to both *E. coli* HPI^+^ and ΔHPI strains, without affecting *Akt* or *mTOR* mRNA levels (Figure A5A). Immunofluorescence staining confirmed the transcriptional suppression of *Beclin-1* and *LC3* at the protein expression. Specifically, strong *Beclin-1* upregulation was observed in *E. coli* HPI^+^-infected control cells at 8 h post-infection, which was effectively abolished in *Beclin-1* depleted cells (Figure 7A,B). Similarly, LC3 immunostaining, which was markedly elevated in *E. coli* HPI^+^-infected cells, was significantly attenuated upon *Beclin-1* knockdown in both bacterial backgrounds, though the HPI^+^ + si*Beclin-1* group still exhibited higher LC3 than its ΔHPI + si*Beclin-1* counterpart (Figure 7C,D). We further investigated whether autophagy deficiency influenced inflammatory output. ELISA and RT-qPCR analyses showed that *E. coli* HPI^+^ infection enhanced both mRNA (by RT-qPCR) and protein (by ELISA) levels of *IL-1β* and *TNF-α* in a time-dependent manner. *Beclin-1* knockdown further amplified cytokine production in infected cells, suggesting that autophagy deficiency may exacerbate infection-triggered inflammation (Figure 7E). To assess the functional consequence of *Beclin-1* loss on cellular integrity, we performed H&E staining of RAW264.7 cells. *E. coli* HPI^+^ infection induced more severe cytopathology including cell rounding, nuclear condensation, and fragmentation than the *E. coli* ΔHPI strain. *Beclin-1* knockdown further exacerbated these morphological alterations, with enhanced nuclear dissolution and cytoplasmic pallor evident in both infection groups, consistent with autophagy deficiency aggravating infection-induced cellular damage (Figure A5B).

## 4. Discussion

Autophagy plays a complex and context-dependent role in maintaining cellular homeostasis [22]. It serves as a surveillance mechanism that protects cells from functional decline by removing damaged organelles and protein aggregates, thereby limiting dysfunctional mitochondria, reactive oxygen species, and DNA damage [23]. In addition, autophagy contributes to innate and adaptive immunity and helps prevent the emergence and persistence of dysregulated cell populations [24]. Although HPI-bearing *E. coli* has been shown to induce autophagy in macrophages, as reported by Zhao et al. The functional role of this pathway in host defense and its underlying regulatory mechanisms remain poorly defined and have been inconsistently reported [25]. Here, we demonstrate that HPI enhances infection-induced autophagic activation in host cells, as evidenced by increased LC3 puncta, upregulation of *Beclin-1* and autophagy-related gene expression, and accumulation of autophagosomes observed by transmission electron microscopy. Notably, this autophagic activation was accompanied by reduced expression of *PI3K*, *Akt*, and *mTOR* at both transcriptional levels, suggesting that HPI may promote autophagy, through downregulation of this pathway. However, despite this autophagic response, HPI infection concurrently exacerbated intestinal inflammation and tissue injury, indicating that the endogenous autophagic response is insufficient to fully counteract HPI-mediated pathogenesis. This sets the stage for exploring whether pharmacological modulation of autophagy could overcome this limitation.

Beyond the core autophagic machinery, multiple signaling pathways have been implicated in the regulation of autophagy during bacterial infection, including AMPK [26], MAPK [27], ER stress [28], NF-κB [29], and reactive oxygen species [30]. The energy sensor AMPK, upon activation under energy stress, suppresses mTORC1 activity via TSC1/TSC2 and promotes autophagy. The MAPK family members ERK1/2, p38, and JNK have also been shown to participate in autophagic regulation, with distinct and sometimes opposing effects depending on cell type and stimulus. Additionally, ER stress, NF-κB, and ROS are known to modulate autophagy in the context of bacterial infections. While these pathways may contribute to the autophagic responses observed in our study, the present study focused on the PI3K/Akt/mTOR axis, a well-established negative regulator of autophagy. The PI3K/Akt/mTOR signaling pathway acts as a master negative regulator of autophagy, functioning as a central “brake system” that suppresses autophagic activity under nutrient-rich conditions [31]. Upon activation by growth factors, PI3K initiates downstream signaling, leading to Akt activation, which subsequently stimulates mTORC1 to inhibit autophagy by phosphorylating key initiation components. The regulatory role of this pathway is commonly investigated using specific pharmacological inhibitors: LY and 3-MA, which target PI3K at early stages of autophagy; TCN, an Akt inhibitor; and rapamycin, which directly inhibits mTORC1 to induce autophagy. By contrast, CQ inhibits autophagic flux at late stages by impairing lysosomal acidification [32]. In our experimental setting, rapamycin alleviated HPI-induced pathology. However, given its broad immunological effects beyond autophagy induction, the clinical potential of rapamycin or its derivatives should be interpreted with caution. Further studies using more specific autophagy inducers or tissue-targeted approaches are warranted to validate the therapeutic translatability.

In addition to its role in autophagy, mTORC1 also controls protein synthesis and cellular metabolism via its downstream effectors p70S6K and 4EBP1, both well-established markers of mTOR activity [33]. The apparent discrepancy between reduced PI3K/Akt/mTOR expression and the upregulated mRNA levels of certain autophagy-related genes likely reflects distinct regulatory mechanisms operating at different timescales. Transcriptional changes represent a slower, compensatory cellular response, whereas post-translational modifications provide rapid signal integration [34]. In macrophages, the early transcriptional upregulation of *PI3K*, *Akt*, and *mTOR* likely represents a feedback response to HPI mediated suppression at the protein level, suggesting that HPI primarily acts at the transcriptional or post-transcriptional level rather than solely through acute signaling events. The differential responses observed between intestinal tissue and macrophages may reflect cell type-specific regulatory dynamics, but the net outcome across both models is consistent: HPI is associated with reduced expression of *PI3K*, *Akt*, and *mTOR*, which in turn relieves the inhibitory constraint on autophagy. Consistent with the central role of mTOR as a gatekeeper of autophagy, its downregulation at both transcriptional and protein levels is associated with autophagic activation. In mice infected with *E. coli* HPI, the downregulation of mTOR could potentially blunt p70S6K signaling and relieve 4EBP1-mediated inhibition, consistent with our transcriptional data showing reduced *p70S6K* and increased *4EBP1* mRNA levels in the mouse intestine (Figure 1). This pattern suggests that HPI-mediated suppression of the PI3K/Akt/mTOR axis may attenuate p70S6K-mediated negative feedback, potentially sustaining autophagic induction and creating a feed-forward loop that amplifies autophagy during infection.

The core machinery of autophagy consists of a set of evolutionarily conserved ATG proteins that act as the essential “builders” of autophagosomes [35]. Beclin-1 is a major regulatory node that integrates upstream signals to modulate autophagy. While Beclin-1 functions as a core component of the Class III PI3K (VPS34) complex to generate PI3P for autophagosome nucleation, Class I PI3K mediates growth factor signaling to activate Akt/mTOR and suppress autophagy. PI3P serves as a membrane-associated protein scaffold required for multiple cellular events. PI3K complex I generates PI3P essential for macroautophagy, whereas PI3K complex II produces PI3P required for endosomal sorting complexes required for transport (ESCRT)-mediated multivesicular body (MVB) formation [36]. This observation is consistent with the known role of Beclin-1 in modulating growth factor receptor signaling via its control of early endosome maturation, as demonstrated by Rohatgi et al. [37], suggesting that Beclin-1 may influence *PI3K* gene expression through feedback regulation. Reduced Beclin-1 expression delays early endosome maturation, which prolongs growth factor receptor signaling and may, in turn, transcriptionally upregulate PI3K as part of a compensatory response. In contrast, *Akt* and *mTOR* mRNA levels remained unchanged, consistent with these kinases being primarily regulated at the post-translational level. This selective transcriptional effect is consistent with a potential regulatory connection between Beclin-1 and Class I *PI3K* gene expression, which may operate independently of the broader PI3K/Akt/mTOR signaling cascade. The conjugation systems involving ATG5, ATG7, ATG16L1, and ATG3 are central to autophagosome membrane elongation [38]. ATG7 functions as an E1-like activating enzyme, facilitating the conjugation of ATG12 to ATG5. The resulting ATG12-ATG5 complex then associates with ATG16L1 to form an E3-like ligase complex essential for LC3 lipidation [39]. LC3 is a widely recognized marker of autophagosomes. In our study, infection with *E. coli* HPI^+^ increased the expression of autophagy-related genes at early time points. Elevated levels of Beclin-1, LC3, and ATG16L1 were detected by immunofluorescence, along with an increase in LC3 puncta formation, suggesting enhanced autophagosome synthesis. Furthermore, comparison between the HPI^+^ and ΔHPI groups revealed that the presence of HPI^+^ further potentiated the upregulation of autophagy-related genes and proteins induced by pathogenic *E. coli* infection. Our results suggest that HPI infection elicits autophagic activation. However, the degree of autophagy induced by HPI alone is insufficient to fully counteract the direct pro-inflammatory and cytotoxic effects of the pathogen because HPI^+^ infection still caused more severe tissue damage than the ΔHPI strain. Importantly, when autophagy was pharmacologically enhanced by rapamycin, the HPI-induced injury was significantly alleviated, whereas autophagy inhibition by 3-MA exacerbated it. These observations support a model in which HPI’s intrinsic virulence overrides its autophagic effects, but tipping the balance toward stronger autophagy can rescue the host from excessive damage.

The ultrastructural abnormalities observed by TEM, particularly the presence of “dumbbell-shaped” and “vacuolated” mitochondria, are strongly suggestive of mitochondrial clearance, the selective removal of damaged mitochondria [40]. Mitochondrial fission, which generates smaller mitochondrial fragments, is a well-established prerequisite for this process, as it facilitates the engulfment of damaged organelles by autophagosomes [41]. The “vacuolated” mitochondria likely represent an intermediate stage of mitochondrial degradation, where damaged organelles are sequestered within autophagic vesicles en route to lysosomal clearance. We propose that HPI-induced mitochondrial damage triggers this compensatory response aimed at eliminating dysfunctional mitochondria. However, as with the autophagic response, this removal process appears insufficient to fully counteract the extent of HPI-induced mitochondrial injury, which may contribute to the exacerbated cellular damage and inflammation observed in HPI^+^-infected cells. This interpretation is consistent with the findings of Roxas et al., who showed that enteropathogenic *E. coli* infection can induce mitochondrial fragmentation and mitophagy [42].

In addition to its effects on autophagy, HPI also modulated mucosal immune responses. M cells are specialized epithelial cells residing in the follicle-associated epithelium overlying Peyer’s patches and isolated lymphoid follicles; they sample and transport luminal antigens for immune surveillance [43]. M-cell differentiation and maturation depend on RANKL expression, which induces the transcription factor Spi-B in crypt-based immature epithelial cells, driving their differentiation into M cells [44]. Accordingly, RANKL is required to sustain both M cell differentiation and functional maturation. M cells sample antigens to initiate mucosal immunity and thereby support IgA production by plasma cells in the lamina propria [45]. The resulting dimeric IgA is transcytosed to the gut lumen, where it is released as sIgA bound to the secretory component derived from proteolytically cleaved p-IgR. This system directly links M-cell activity to luminal immune surveillance, making sIgA concentration a reliable marker of mucosal immune status [46]. The HPI-mediated increase in M-cell differentiation presents a functional dichotomy. M cells serve as the primary portal for luminal antigen sampling and are exploited by numerous enteric pathogens, including *Salmonella*, *Shigella*, and *Yersinia*, to breach the intestinal epithelial barrier [47]. In this context, HPI-mediated RANKL upregulation acts as a double-edged mechanism. Enhanced M-cell numbers may strengthen immune surveillance, consistent with the elevated sIgA response observed following HPI^+^ infection. Conversely, the expansion of these transcytotic portals facilitates bacterial translocation, amplifies local inflammation, and exacerbates intestinal tissue injury. These findings indicate that HPI functionally co-opts the host RANKL-M cell axis to promote its own pathogenesis, shifting the balance from mucosal defense toward bacterial dissemination and tissue damage [48]. We acknowledge that intraperitoneal (i.p.) infection does not recapitulate the oral route and is therefore a limitation. However, the model was chosen for its utility in controlled virulence assessment, and systemic infection can still trigger mucosal responses via the common mucosal immune system [49]. Consistent with this, sIgA levels were consistently lower after infection with the ΔHPI strain than with the HPI^+^ strain, suggesting that HPI promotes a stronger mucosal immune response. Oral infection models would be more physiologically relevant for intestinal mucosal studies and should be pursued in future work.

Cytokines are key signaling molecules that coordinate immune responses. Upon exposure to inflammatory stimuli, macrophages secrete a range of pro-inflammatory cytokines, including IL-1β, IFN-γ, and TNF-α [50]. In the intestinal mucosa, resident macrophages contribute to host defense by limiting pathogen entry and colonization [51]. During intestinal inflammation, inflammatory macrophages are sequentially recruited to mount immune responses and amplify the production of pro-inflammatory cytokines [52]. Importantly, macrophage autophagy deficiency can promote intracellular bacterial survival and exacerbate pro-inflammatory cytokine secretion. In vivo, infection with *E. coli* HPI^+^ increased intestinal *TNF-α*, *IL-1β*, and *IFN-γ* expression to levels significantly higher than those induced by the ΔHPI strain. In vitro, *IL-1β* expression was significantly lower in response to *E. coli* HPI^+^ than to the ΔHPI strain, whereas *TNF-α* and *IFN-γ* showed similar elevation (Figure 6). This discrepancy in IL-1β levels likely reflect the lack of a coordinated immune environment in vitro. The intestinal microenvironment provides continuous TLR priming signals that sustain high basal pro-IL-1β in tissue macrophages, whereas cultured cells lack such cues [53]. Under these conditions, HPI may activate the inflammasome in vivo but potentially inhibit its function in vitro when priming is insufficient. Alternatively, HPI may act through autophagy-related pathways that operate distinctly in isolated cells. Our observations regarding HPI-mediated changes in the PI3K/Akt/mTOR pathway are based on comparisons between the *E. coli* HPI^+^ and *E. coli* ΔHPI strains. Given that HPI comprises multiple genes involved in iron acquisition, metabolism, and bacterial fitness, the specific HPI-encoded factor responsible for the observed effects on host autophagy and inflammatory responses remain unclear. While our data show an association between the presence of HPI and altered host responses, they do not establish a direct causal relationship attributable to a single HPI gene. Future studies employing targeted deletions of individual HPI genes, particularly those encoding the yersiniabactin siderophore system, will be necessary to dissect the causal relationship between HPI components and the autophagic phenotypes observed here.

## 5. Conclusions

Our findings suggest that the presence of HPI in a Saba pig-derived *E. coli* strain correlates with enhanced intestinal inflammation and pathology, increased M cells differentiation and sIgA transcytosis, and elevated autophagic activity associated with reduced expression of *PI3K*, *Akt* and *mTOR*. Notably, pharmacological enhancement of autophagy with rapamycin alleviated HPI-associated tissue damage, whereas autophagy inhibition by 3-MA exacerbated it, indicating that while HPI induces an autophagic response, this endogenous response is insufficient to fully counteract HPI-mediated pathogenesis. Overall, these data suggest that HPI may contribute to bacterial virulence and intestinal injury through direct pro-inflammatory effects, while the concurrent autophagic response partially limits excessive tissue damage only when pharmacologically enhanced. However, as the HPI consists of multiple genes involved in iron acquisition and bacterial fitness, further studies using targeted gene deletions are required to identify the specific HPI-encoded effector responsible for the observed phenotypes.

## Figures and Tables

**Table 1:** Sequences of primers used for RT-qPCR in this study.

Gene	Forward Primer (5′ → 3′)	Reverse Primer (5′ → 3′)
*β-actin*	TGCGGGACATCAAGGAGA	AGGAAGGAGGGCGGAAGAG
*RANKL*	CCATCGGGTTCCCATAAAGTCAGT	AAAGCCCCAAAGTACGTCGCATCT
*PI3K*	AATGCACGGCGATTACACTC	GGACACTGGGTAGAGCAACT
*Beclin-1*	AGGAGCTGCCGTTGTACTGTTCT	TGCTGCACACAGTCCAGGAA
*LC3*	AACGAAATTCCTGGTGCCTGA	AAGGCTTGGTTAGCATTGAGCTG
*Akt*	CTGCCCTTCTACAACCAGGA	CATACACATCCTGCCACACG
*mTOR*	GCCACTCTCTGACCCAGTTC	GGTTATCCCAACCACGAGCA
*ATG3*	ACACGGTGAAGGGAAAGGC	TGGTGGACTAAGTGATCTCCAG
*ATG5*	TGCGGTTGAGGCTCACTTTA	GTTGATGGCCCAAAACTGGT
*ATG7*	GTTCGCCCCCTTTAATAGTGC	TGAACTCCAACGTCAAGCGG
*4EBP1*	GGGGACTACAGCACCACTC	CTCATCGCTGGTAGGGCTA
*P70S6K*	AAATCCGATCGCCTCGAAGA	CACTTGTTTCCATTGGGTATTCCAC
*TNF-α*	CCCTCACACTCAGATCATCTTCT	GCTACGACGTGGGCTACAG
*IFN-γ*	ATGAACGCTACACACTGCATC	CCATCCTTTTGCCAGTTCCTC
*IL-1β*	GCAGTGGAGAAGCCGATGA	GGTGGAGAGCCTTCAGCAT

## Data Availability

The data that support the findings of this study are openly available in Mendeley Data at https://doi.org/10.17632/mb9srn6tgb.1.

## Abbreviations

The following abbreviations are used in this manuscript:

*E. coli*	*Escherichia coli*
HPI	High-pathogenicity island
ATG	Autophagy-related genes
LC3	light chain 3
mTOR	mammalian target of rapamycin
PI3K	phosphatidylinositol 3-kinase
Akt	Protein Kinase B
M cell	Microfold cell
TNF-α	Tumor necrosis factor alpha
IFN-γ	Interferon-gamma
TEM	transmission electron microscopy
3-MA	3-Methyladenine
IgA	immunoglobulin A
IL-1β	Interleukin-1β
H&E	Hematoxylin & Eosin Staining
MOI	Multiplicity of infection
CQ	Chloroquine
LY	LY294002
TCN	Triciribine
LDH	lactic dehydrogenase
